# Understanding climate change impacts on biome and plant distributions in the Andes: Challenges and opportunities

**DOI:** 10.1111/jbi.14389

**Published:** 2022-06-03

**Authors:** Carolina Tovar, Andrea F. Carril, Alvaro G. Gutiérrez, Antje Ahrends, Lluis Fita, Pablo Zaninelli, Pedro Flombaum, Ana M. Abarzúa, Diego Alarcón, Valeria Aschero, Selene Báez, Agustina Barros, Julieta Carilla, M. Eugenia Ferrero, Suzette G. A. Flantua, Paúl Gonzáles, Claudio G. Menéndez, Oscar A. Pérez‐Escobar, Aníbal Pauchard, Romina C. Ruscica, Tiina Särkinen, Anna A. Sörensson, Ana Srur, Ricardo Villalba, Peter M. Hollingsworth

**Affiliations:** ^1^ Royal Botanic Gardens Kew Surrey UK; ^2^ Universidad de Buenos Aires – CONICET Centro de Investigaciones del Mar y la Atmósfera (CIMA) Buenos Aires Argentina; ^3^ CNRS – IRD – CONICET – UBA Institut Franco‐Argentin d'Études sur le Climat et ses Impacts (IFAECI) Buenos Aires y Mendoza Argentina; ^4^ Departamento de Ciencias Ambientales y Recursos Naturales Renovables, Facultad de Ciencias Agronómicas Universidad de Chile Santiago Chile; ^5^ Instituto de Ecología y Biodiversidad (IEB) Chile; ^6^ Royal Botanic Garden Edinburgh Edinburgh UK; ^7^ Universidad Nacional de La Plata, La Plata Facultad de Ciencias Astronómicas y Geofísicas La Plata Argentina; ^8^ Universidad de Buenos Aires Facultad de Ciencias Exactas y Naturales Departamento de Ecología, Genética y Evolución Buenos Aires Argentina; ^9^ Universidad Austral de Chile Instituto Ciencias de la Tierra Valdivia Chile; ^10^ Instituto Argentino de Nivología Glaciología y Ciencias Ambientales (IANIGLA), CCT‐CONICET Mendoza Argentina; ^11^ Universidad Nacional de Cuyo Facultad de Ciencias Exactas y Naturales Mendoza Argentina; ^12^ Departamento de Biología Escuela Politécnica Nacional del Ecuador Quito Ecuador; ^13^ Instituto de Ecología Regional Universidad Nacional de Tucumán – CONICET Tucumán Argentina; ^14^ Laboratorio de Dendrocronología Universidad Continental Huancayo Peru; ^15^ Department of Biological Sciences University of Bergen Bergen Norway; ^16^ Bjerknes Centre for Climate Research University of Bergen Bergen Norway; ^17^ Laboratorio de Florística, Departamento de Dicotiledóneas Universidad Nacional Mayor de San Marcos, Museo de Historia Natural Lima Peru; ^18^ Departamento de Ciencias de la Atmósfera y los Océanos, Facultad de Ciencias Exactas y Naturales Universidad de Buenos Aires Buenos Aires Argentina; ^19^ Laboratorio de Invasiones Biológicas (LIB), Facultad de Ciencias Forestales Universidad de Concepción Concepción Chile

**Keywords:** Andes, climate change, plant biodiversity, plant dynamics, species distribution modelling

## Abstract

**Aim:**

Climate change is expected to impact mountain biodiversity by shifting species ranges and the biomes they shape. The extent and regional variation in these impacts are still poorly understood, particularly in the highly biodiverse Andes. Regional syntheses of climate change impacts on vegetation are pivotal to identify and guide research priorities. Here we review current data, knowledge and uncertainties in past, present and future climate change impacts on vegetation in the Andes.

**Location:** Andes.

**Taxon:** Plants.

**Methods:**

We (i) conducted a literature review on Andean vegetation responses to past and contemporary climatic change, (ii) analysed future climate projections for different elevations and slope orientations at 19 Andean locations using an ensemble of model outputs from the Coupled Model Intercomparison Project 5, and (iii) calculated changes in the suitable climate envelope area of Andean biomes and compared these results to studies that used species distribution models.

**Results:**

Future climatic changes (2040–2070) are projected to be stronger at high‐elevation areas in the tropical Andes (up to 4°C under RCP 8.5), while in the temperate Andes temperature increases are projected to be up to 2°C. Under this worst‐case scenario, temperate deciduous forests and the grasslands/steppes from the Central and Southern Andes are predicted to show the greatest losses of suitable climatic space (30% and 17%–23%, respectively). The high vulnerability of these biomes contrasts with the low attention from researchers modelling Andean species distributions. Critical knowledge gaps include a lack of an Andean wide plant checklist, insufficient density of weather stations at high‐elevation areas, a lack of high‐resolution climatologies that accommodates the Andes' complex topography and climatic processes, insufficient data to model demographic and ecological processes, and low use of palaeo data for distribution modelling.

**Main conclusions:**

Climate change is likely to profoundly affect the extent and composition of Andean biomes. Temperate Andean biomes in particular are susceptible to substantial area contractions. There are, however, considerable challenges and uncertainties in modelling species and biome responses and a pressing need for a region‐wide approach to address knowledge gaps and improve understanding and monitoring of climate change impacts in these globally important biomes.

## 1 INTRODUCTION

**FIGURE 1:**
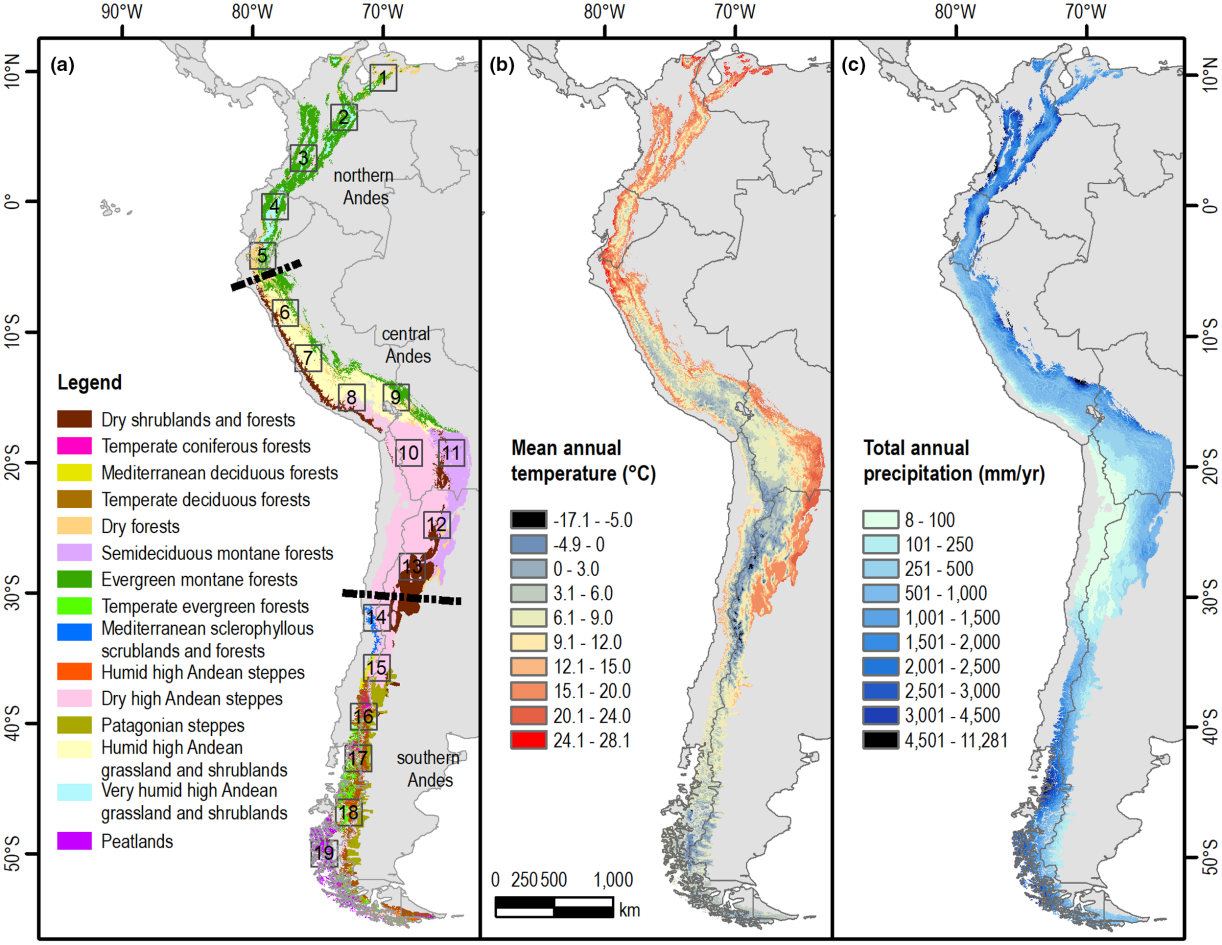
The Andes. (a) Andean biomes based on three vegetation maps (Luebert & Pliscoff, 2018; Oyarzabal et al., 2018; Tovar et al., 2013), major three regions (Northern, Central and Southern Andes) and 19 locations (2° × 2° bounding boxes, indicated by the numbers along the Andes) where climate change projections were analysed for this review, (b) mean annual temperature and (c) total annual precipitation obtained from CHELSA for the period 1979–2013 (Karger et al., 2017). Maps in geographical coordinate system

The Andes (Figure 1) are among the most biodiverse regions on the planet (Myers et al., 2000). Spanning over 9000 km in length (following the mountain ridge) (Graham, 2009) and reaching well over 6000 m in elevation, they are the longest and second highest terrestrial mountain range on Earth after the Himalayas. The Andes are home to an estimated 40,000 plant species and thousands of vertebrate species with exceptionally high levels of species endemism (Kreft & Jetz, 2007; Pennington et al., 2010). In addition, their natural forests, shrublands and grasslands provide critical ecosystem services such as soil protection, carbon storage and—notably—water for millions of people (e.g. Buytaert et al., 2011; Diazgranados et al., 2021; Masiokas et al., 2019; Peña et al., 2018). Understanding the links between climate and natural vegetation, and predicting the impact of future climate change, is thus important for both conservation and human well‐being in the Andes and adjacent lowlands. Given the long latitudinal and steep elevational range, the Andes have a high variability in climate (e.g. Espinoza et al., 2020; Pabón Caicedo et al., 2020) (Figure 1b,c), making them an ideal natural laboratory for studying climate change impacts on plant biodiversity along these gradients.

In the Andes, there are significant knowledge gaps and uncertainties surrounding the effects of climate change on the distribution of species and biomes. Recent papers have contributed to a greater understanding of the topic (e.g. Blundo et al., 2012; Carilla et al., 2018; Duque et al., 2015; Feeley et al., 2011; Srur et al., 2016), but broad‐scale overview studies of the Andes are lacking. Here, we review available data and knowledge on the likely impacts of climate change on plant and biome distributions in the region. Specifically, we combine (i) a literature review on past (long term, medium term and short term) climate change impacts on Andean vegetation, with (ii) an analysis of the outputs of global climate models to characterise future projections for the region and associated uncertainties, and (iii) modelling the potential impacts on the distribution of Andean biomes. Finally, we take a critical look at the limitations of current distribution modelling practice and propose a research agenda to fill knowledge gaps to enable targeted conservation action for plants and biomes in the Andes.

## 2 MATERIALS AND METHODS

### 2.1 Literature review: Past climate and vegetation changes

We conducted a literature review of studies on long‐term (thousands of years) and medium‐ to short‐term (last millennium and decades) past climate changes and vegetation responses. Knowledge on past vegetation dynamics over long time‐scales is based on palaeoecological research and specifically fossil pollen records. Around 1650 of such records are known to exist across Latin America based on over 1700 studies (See inventory of fossil pollen records by Flantua et al., 2015 and www.latinamericanpollendb.com) which we complemented with additional studies published between 2015 and 2016. We selected only records located within the Andes (*n* = 742) using the shapefile of the limits of the Andean biomes (see Section 2.4). In our results, we summarised the main vegetation responses to long‐term climate change based on the spatial coverage of these records.

Medium‐ and short‐term changes were analysed and summarised through a systematic literature review. We conducted a search for peer‐reviewed articles on Scopus on 1st of April 2021 using the following keywords ‘Andes’ or ‘Patagonia’, ‘climate change’ or ‘drought’ or ‘deglaciation’, ‘tree’ or ‘vegetation’ or ‘plant’, and ‘chronosequence’ or ‘plots’ or ‘resurvey’ or ‘tree‐ring’ or ‘dendrochronological’. This allowed us to source publications covering the entire Andes, the main climate changes and the main methods used to analyse vegetation changes over this time‐scale. We obtained 131 studies that were checked to keep only those that recorded changes in climate for a given period and/or a vegetation response to past climate change. We also kept those related to fire events linked to climate conditions. Finally, 58 studies were used (Table S1) to characterise medium‐ and short‐term changes.

### 2.2 Literature review: Plant distribution modelling

Two main approaches are currently used to model plant species distributions, namely correlative and dynamic models. Correlative models, commonly known as species distribution models (SDMs) or ecological niche models, relate species presence at a certain location to environmental conditions using an algorithm (Guisan & Thuiller, 2005). In contrast, Dynamic Vegetation Models (DVMs) are based on a mechanistic approach, and are able to reflect demographic and ecological processes shaped by physiological constraints and species competition, among others (Guisan & Thuiller, 2005; Snell et al., 2014).

We performed a literature review of plant distribution modelling in the Andes, using either approach. For correlative models, we conducted a search in Scopus (29th of January 2020) using the keywords ‘climate change’ or ‘warming’ and ‘species distribution’ and retrieved 145 publications for 2010–2019. Separate queries were conducted for each Andean country and one additional query used the keywords ‘Andes’ or ‘Andean’ and ‘plant’. After examining all papers, we kept studies that used SDMs to model present, past and/or future distributions of Andean plant species with a final selection of 32 studies (Table S2), while a much lower number of studies used Dynamic Vegetation Models.

### 2.3 Future climate change projections

To assess projected future climatic change in the Andes, we used an ensemble of model outputs from the Coupled Model Intercomparison Project 5 (CMIP5, https://www.wcrp‐climate.org/wgcm‐cmip/wgcm‐cmip5; Table S3). For this, we selected 19 locations across the Andes, each encompassing a 2° × 2° bounding box covering an area of ~40,000–50,000 km^2^ each (Table S4; Figure 1a). As the mountain range blocks atmospheric circulation, projected shifts in climate vary not only at different elevations, but also between western and eastern slopes (Arias et al., 2021). Therefore, we used a novel approach to quantify and analyse projected changes in climate along slopes, differentiating areas based on their topography. Annual mean precipitation and near‐surface air temperature data from the CMIP5 models were grouped within each location according to elevation range (discretised by 500 m intervals) and aspect (western slope, peak and eastern slope). In this way, data from different models are only combined if they belong to a given aspect and to the same elevation range (i.e. at each elevation‐aspect combination we got a different ensemble with a different number of contributing models). Finally, since the grid cells of the models only partially coincide with the 2° x 2° bounding box, a weighted average was performed taking into account the percentage of the bounding box covered by each grid cell. In this procedure, the data from the different models are not interpolated to a common grid projection and resolution (see Methods S1 for details). This method was presented by Fita et al. (2019) and was previously used by Pabón‐Caicedo et al. (2020).

Changes in climate were calculated as the ensemble mean difference between projected future (2040–2070, most extreme scenario RCP 8.5) and near‐present conditions (1960–1990, ‘historical scenario’). Scenario RCP 8.5 assumes a drastic increase in the use of coal and was designed to simulate an extreme non‐mitigation situation with increasing population and energy demand. While policy endeavours render this scenario increasingly unlikely, it can be used to understand what could happen in the worst case and which systems/biomes would be potential beneficiaries and where one would expect to see drastic losses. Albeit drastic, RCP 8.5 is not implausible (Schwalm et al., 2020), and it is useful to understand its implication. The robustness of the changes between current and worst‐case future conditions was assessed using the signal‐to‐noise ratio (SNR) (Kendon et al., 2008), which is a measure of models agreement, usually called ‘spread’ (see also details in Methods S2).

### 2.4 Future projections of vegetation responses

To assess potential climate change impacts on Andean biomes, we first created a unified high‐resolution biome map covering the entire Andean region by combining existing vegetation maps (Luebert & Pliscoff, 2018; Oyarzabal et al., 2018; Tovar et al., 2013). To standardise these maps with different levels of class resolution, we merged several classes to delineate standardised main functional types throughout the Andes. In total, we delineated 15 biomes/vegetation types (Table S5) based on dominant plant functional types (e.g. evergreen/deciduous, tree/shrubs) and dominant climate (e.g. dry, humid). Before assessing potential future changes in the climate envelops of these biomes, we first characterised their current climate envelope using annual mean temperature and total annual precipitation following the widely accepted Whittaker's classification (Whittaker, 1975). For this, we extracted climate data from CHELSA at a resolution of 10 arc minutes (≈18.5 km; Karger et al., 2017) to obtain a higher resolution than the one provided by CMIP5. Then, we applied a simplified delta method (Hay et al., 2000) where the differences in climate obtained from CMIP5 model outputs are added to the observed values from CHELSA for each biome. For each CMIP5 model (Table S3), we estimated the ‘delta‐change’ for the mean annual temperature and total annual precipitation data between the future and present at their original resolution. We used for future climate the 2040–2070 period from the RCP8.5 scenario and the 1960–1990 period as the near‐present climate (‘historical scenario’). Then, the deltas obtained from CMIP5 models were applied to the current climate of each biome (characterised from the CHELSA data) at grid point basis, to estimate their projected changes for the whole Andean region. Results were expressed as relative changes in the extent covered by the present‐day climatic envelope of each biome and assessed using the SNR (Kendon et al., 2008).

## 3 PAST VEGETATION CHANGE IN THE ANDES

**FIGURE 2:**
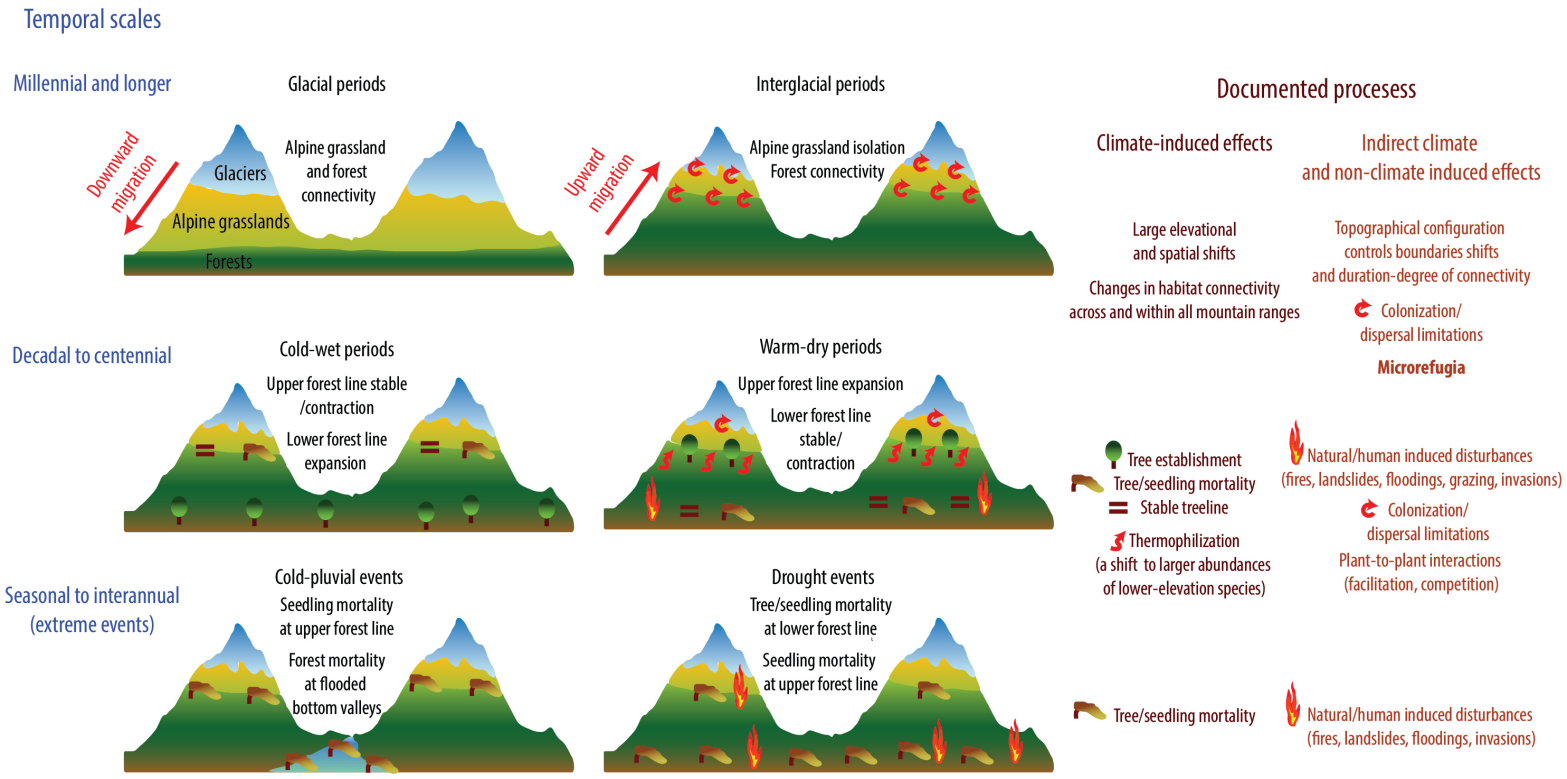
Representation of vegetation responses to past changes in climate at different temporal scales identified in palaeoecological records and plot data from the Andes

An understanding of how past climatic changes shaped Andean vegetation can inform how biomes might respond to projected future climate. Here, we summarise general findings from palaeoecological studies that cover the last glacial–interglacial cycle (last c. 120,000 years), based on fossil pollen records, and mid‐ and short‐term studies, based on dendrochronology and monitoring (Figure 2).

### 3.1 Long‐term changes

Millions of years of geological processes have led to the rise of the Andes, a mountain range that has determined the geography, geomorphology and climate of the whole of South America (Boschman, 2021; Ehlers & Poulsen, 2009; Insel et al., 2010) and the Southern Hemisphere (Falco et al., 2019). Moisture fluxes are concentrated, deviated or blocked along the longitudinal and latitudinal shape of this stretched mountain system (Garreaud, 2009). The spatial and temporal patterns of the Andean climate are affected by ocean–atmosphere interactions, monsoon systems, the seasonal migrations of the Intertropical Convergence Zone (ITCZ) over the Atlantic and Pacific oceans, the southern westerly wind belt (SWW) and storm track at mid and high latitudes (Arias et al., 2021; Garreaud et al., 2009), jointly causing climate variability on interannual to interdecadal scales (e.g. Flantua et al., 2016).

The intensity and mean latitude of the ITCZ, atmospheric convective systems and the trade winds were influenced by Quaternary (last 2.6 million years) climate changes, causing changes in precipitation regimes over South America and especially along the eastern tropical Andes (Novello et al., 2017, 2019). In the Southern Andes, the changes in the intensity and latitudinal position of the SWW caused strong variations in rainfall during the last glacial–interglacial cycle (Lamy et al., 2010; Rojas et al., 2009). Paleotemperatures have been recorded on glacier snowline reconstructions and fossil pollen records during the Quaternary, evidencing a remarkable variability of geo‐climatic scenarios. For example, during the last glacial–interglacial cycle, several periods of relatively warmer climates (interstadials) were interrupted by periods of cooler climates (stadials) with sharp temperature declines during glacial advances in the Northern (Groot et al., 2013) and Southern (Kaiser et al., 2005; Villagrán et al., 2019) Andes. Temperature ranges over a full glacial–interglacial cycle could have reached 5–10°C in the high Northern and Central Andes above 2500 m (e.g. Flantua et al., 2019; Groot et al., 2011; Hooghiemstra & Flantua, 2019; Klein et al., 1995; Mark et al., 2005; Valencia et al., 2010), while at mid‐latitudes of the Southern Andes, a decrease in mean summer temperatures of 6–8°C below modern values has been estimated (e.g. Heusser et al., 1981). Temperature estimates of periods warmer than present, such as the early to mid‐Holocene climatic optimum (c. 10,000–6000 years ago depending on the region) and the last interglacial are scarce, but along the coast of the Central and Southern Andes, for instance, temperature is estimated to have been 3–4°C higher than today (Heusser et al., 1981; see more in Mayle et al., 2004).

Fossil pollen records have contributed to understanding biome responses to long‐term paleoclimate fluctuations at long and short time‐scales. While there is a relatively high density of fossil pollen records for the Northern Andes and the tip of the Patagonian Andes (Figure S1), only few records cover the last glacial–interglacial cycle as most fossil pollen records reach only the Holocene (last 11,700 years) (Flantua et al., 2015). In the Southern Andes, only few continental records span the last glacial maximum (LGM) and beyond due to the massive extent of glaciers in this part of the Andes (Palacios et al., 2020). The scarce long fossil pollen records from the Northern and Central Andes have, however, provided evidence of the sensitivity of Andean biomes to Quaternary climate fluctuations, responding with elevational shifts of biomes and taxa over long time‐scales (e.g. Hooghiemstra & Flantua, 2019). These elevational shifts caused substantial changes in habitat fragmentation for the high‐elevation grass biome (páramos) of the Northern Andes, likely contributing to the build‐up of its exceptional biodiversity over the course of the Quaternary (Flantua et al., 2019). Andean forests in the Northern Andes, on the other hand, show little change in species composition over long time‐scales, that is, between glacial–interglacial cycles (Felde et al., 2016), likely the effect of a continuous high habitat connectivity unaffected by the Quaternary climate fluctuations (Flantua & Hooghiemstra, 2018).

In the Southern Andes, during the stadials, glacial vegetation north of 42° S in the western Andes was dominated by *Nothofagus* and conifer forests together with Magellanic moorlands (Abarzúa et al., 2014; Villagrán, 2001; Villagrán et al., 2019). In contrast, Patagonia (southern to 42°S) was dominated by a cold and dry steppe vegetation during the glacial period, up to 51,000 years BP (Recasens et al., 2012). During the warmer periods, the *Nothofagus* and coniferous forests expanded further south and to lower elevations in the western cordillera (Heusser et al., 1981; Villagrán et al., 2019). Today, these forests exist only as relicts at mountain summits in north Patagonia or in sub‐Antarctic forest formations.

Over shorter time‐scales (last 30,000 years), high‐resolution palaeoecological records provide abundant evidence of gradual replacement and temporal disappearance of taxa in response to shifts in climate conditions (e.g. thermophilisation; Bogotá et al., 2016; González‐Carranza et al., 2012; Groot et al., 2013). Also these records provide evidence of non‐analogue biome composition as the result of altitudinal shifts and mixes of high‐ and low‐land taxa in response to climate change (e.g. in eastern forest of the Central Andes, Mayle et al., 2004, 2009) and the presence of microrefugia (e.g. Valencia et al., 2010). At the level of biomes, the spatial distribution differed substantially between the present and that of the LGM at the scale of South America, with altitudinal shifts between forest and grassland biomes in most Andean regions with changes in species composition (Marchant et al., 2009; Mayle et al., 2009; Villagrán, 2001). Under warmer periods than present, there were elevational shifts of biomes to higher elevations. For instance, montane cloud forest expanded upwards in the Northern Andes (Niemann & Behling, 2008). Also, after the glacier retreatment, a mixture of *Nothofagus* forest and shrubland/steppe developed on the east side of the Southern Andes, while temperate rainforests developed on the west (Abarzúa et al., 2004; Whitlock et al., 2006). As temperature increased during the warmer and drier early‐Holocene, fire became an important component of these modern forest biomes in the Southern Andes (e.g. Kitzberger & Veblen, 2003; Whitlock et al., 2006), but also in the high‐elevation grasslands of the Northern and Central Andes (e.g. Villota et al., 2012; Weng et al., 2006).

At millennial time‐scales, Andean biomes have reached their modern diversity and distribution over the late Holocene, according to the increase in climate variability, but also the human impact along the Andes (Armesto et al., 2010; Flantua et al., 2016; Niemann & Behling, 2010).

### 3.2 Mid‐ to short‐term changes

Few high‐resolution regional studies currently exist on vegetation responses to climate changes observed in the last 1000 years (Table S1). Given the heterogeneity in climate and vegetation across the Andes, we briefly discuss reported changes for the Northern, Central and Southern Andes separately.

While positive trends in surface temperatures have been reported across the Andes in the last 100 years, observed trends of precipitation have been both negative and positive (Pabón Caicedo et al., 2020). In Ecuador, one of the oldest climatic datasets shows increasing temperatures since the mid‐1800 (Morueta‐Holme et al., 2015). As a result, an upward shift of 215–266 m in the upper limit of the alpine vegetation has been observed (Moret et al., 2019) in comparison to Humboldt's observations in 1802 (von Humboldt and Bonpland, 1807). Forest vegetation has also shown changes due to increasing temperatures. Thermophilisation, a shift in composition towards greater relative abundances of species from lower and warmer elevations, is reported to be widespread in Northern Andean forests, affecting both adult and juvenile tree communities (Duque et al., 2015; Fadrique et al., 2018). Observed changes seem related to higher than normal tree mortality among cold‐adapted species, which also lead to a decrease in species richness of adult trees (Duque et al., 2015). Such changes in tree mortality due to climate variations have also been observed in fossil pollen records (see e.g. González‐Carranza et al., 2012) and can result in temporal or permanent change in biome composition. The local persistence of species (and hence species richness) is likely to depend on the presence or absence of microrefugia where taxa may reside until climate favours their expansion again, also influenced by the heterogeneity of the Andean ‘mountain fingerprint’ (Flantua & Hooghiemstra, 2018) that may facilitate or inhibit migration along the Andes.

In the Central Andes, positive temperature trends have been reported for example in Peru (Lavado Casimiro et al., 2013; Schauwecker et al., 2014) and Bolivia (Seiler et al., 2014) over the last 100 years. Warming has led to both thermophilisation and primary succession in recently deglaciated areas. Thermophilisation of Peruvian Andean forests at the genus level has been registered over periods of time as short as 4 years (Feeley et al., 2011). Similar results were reported from a network of plots installed across tropical Andean montane forests over the last 15 years (Fadrique et al., 2018). Yet, the rates of thermophilisation are heterogeneous, with areas at intermediate elevations having more species that seem to be less sensitive to temperature increases, possibly due to certain plant traits (e.g. wider thermal tolerances), but also to other factors such as local site conditions (e.g. topgraphy and soil characteristics). Deglaciation has also rapidly increased in the Central Andes since the late 1970s, leading to the formation of new species assemblages (Zimmer et al., 2018). However, succession in response to warming has been slow due to the over‐representation of wind‐dispersed species (initial colonisers) and the low numbers and maturity of nurse plants, whose facilitation role is crucial in alpine ecosystems (Zimmer et al., 2018). Precipitation patterns have been more variable with both negative and positive trends in the last decades. Based on *Polylepis* tree‐ring reconstructions, an unprecedented decrease in precipitation over the past 700 years has been recorded over the arid Altiplano since mid‐20th century (Morales et al., 2012). A similar trend is found in the humid eastern Peruvian Andes (11° S) based on a *Cedrela*‐*J* *uglans* reconstruction that dates to 1817 (Humanes‐Fuente et al., 2020). On the contrary, the northern Argentinian Andes (eastern slope) has experienced sustained increasing precipitation and river streamflow in the last decades, which is unprecedented for the last three centuries (Ferrero et al., 2015; Villalba et al., 1998).

In the Southern Andes, tree‐ring‐based climate reconstructions show a consistent increase in temperature and a marked precipitation decrease in the last century. In the southernmost part of the Andes (47–52° S), tree‐ring chronologies from *Nothofagus pumilio* indicate that over the past 400 years the highest temperatures were found after the beginning of the 20th century (Villalba et al., 2003). Tree rings also reveal unprecedented high summer (December–February) temperatures during the last decades over the last 1000 years in northern Patagonia (37–44°S) (Pabón Caicedo et al., 2020; Villalba, 1990), together with a distinct decrease in precipitation over the last 50 years (Villalba et al., 2012). As a result of the increasing frequency of drought events, tree mortality in the *Nothofagus* forests has risen and tree growth declined since the mid‐1970s, particularly at the lower elevations in the eastern slope of the Andes (Rodríguez‐Catón et al., 2016; Srur et al., 2018; Suarez et al., 2004). In the Mediterranean region of the Chilean Andes (34°S), *Nothofagus macrocarpa* forests show an unprecedent decrease in growth since 1980 compared to the last two centuries (Venegas‐González et al., 2019) as a result of the same climatic trend. Massive mortality events in mesic‐wet forests of *Austrocedrus chilensis* coincided with hot and dry summers in 1912–1913, 1942–1943, 1956 and 1962 (Villalba & Veblen, 1997). Although a substantial *Austrocedrus* establishment occurred during the cool and wet conditions in the region between 1963 and 1979, no new episodes of tree establishment at the lower forest line have been observed since the 1980s, when warmer and drier climatic conditions have prevailed across the Southern Andes. In contrast, the recent warmer temperatures have allowed the establishment of new *Nothofagus pumilio* trees above the upper forest line and on deglaciated terrains across Patagonia (Garibotti et al., 2011; Srur et al., 2018). All the recorded impacts of climate change on tree demographic rates highlight the need to identify the climatic thresholds that regulate establishment and mortality along Andean forests.

## 4 FUTURE CLIMATE CHANGE PROJECTIONS

**FIGURE 3:**
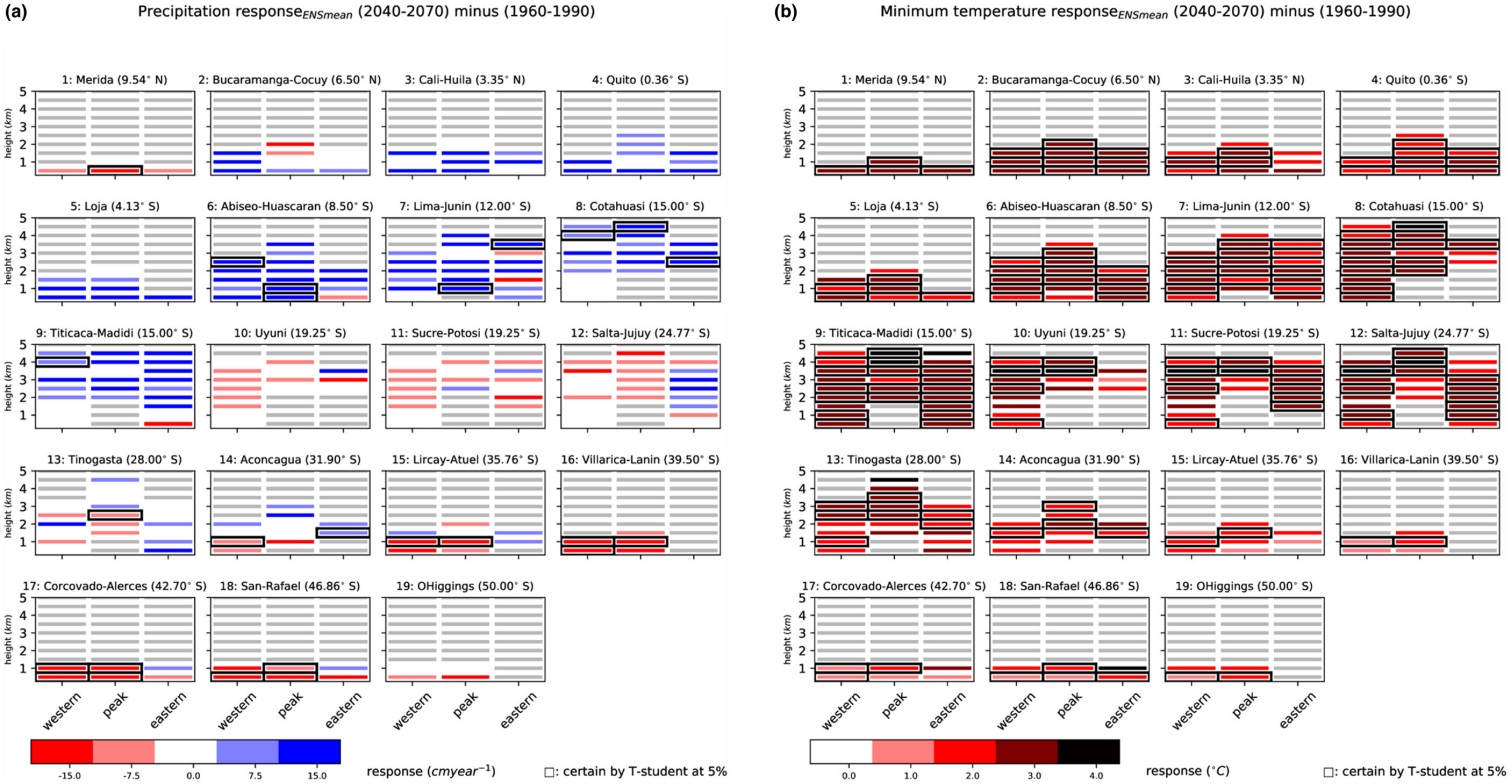
Future projections of precipitation and minimum annual temperature in 19 locations along the Andes indicated in Figure 1a for 2040–2070 RCP8.5. (a) total annual precipitation and (b) annual mean near‐surface air temperature for each location split by 500 m elevation intervals (y‐axis) and aspect (W = western, pk = peak, E = eastern in the x‐axis). The changes are calculated using an ensemble of CMIP5 GCMs, as differences between the future (2040–2070, RCP8.5 scenario) and near‐present conditions (1960–1990). Black edge lines highlight confident changes (SNR at 95%), while grey cells are combinations of elevation and aspect without data. Latitude values represent the geographical coordinates of the top left corner of the bounding box defining each location (see Table S4)

Our results show that while projected future temperatures consistently increase across the Andes, projected changes in precipitation are regionally variable (Figure 3), consistent with Arias et al. (2021). In some regions, total annual precipitation is projected to change by as much as 150 mm. Most regions may experience both increasing and decreasing precipitation depending on slope orientation (Figure 3a). A consistent direction in changes in precipitation across all combinations of elevation and aspect was projected for only a handful of locations, namely in the tropical Andes (e.g. Cali‐Huila, Loja and Quito located between 4.5°N and 3°S and Cotahuasi at 14°S), where precipitation was projected to ubiquitously increase, and in the Southern Andes (e.g. Villarica‐Lanin at 38.5°S and O'Higgins at 49°S) where precipitation was projected to ubiquitously decrease. A few more general patterns emerged: while the Northern‐Central Andes from Cali‐Huila to Titicaca‐Madidi (4.35°N–15°S) may predominantly experience increasing precipitation, areas located in the far north (Mérida; 10.5°N), far south (>39°S) and some areas in the centre (Uyuni to Salta‐Jujuy; 18°S–24°S) may predominantly get drier. However, for most combinations of elevation and aspect, precipitation changes could not be computed with confidence due to the large spread in individual model outputs (Figure 3a).

Both the minimum (Figure 3b) and maximum temperature (Figure S2) were projected to increase across all combinations of elevation and aspect, mostly with high confidence (i.e. with a high signal to noise ratio). The warming trend was slightly stronger for minimum than for maximum temperature. This may be due to the different way in which certain factors, such as cloudiness and increasing water vapour in the atmosphere, alter the surface energy balance during the day (affecting maximum temperature) and during the night (affecting minimum temperature) (IPCC AR4, 2007). Overall, temperature increases are expected to be larger in the Northern and Central Andes than in the Southern Andes. Several factors could be related to this pattern, but the complexity of the South American climate system prevents definite conclusions. One example of this complexity are the different main modes of variability of the climate system affecting the Andes, such as the North Atlantic Oscillation (NAO), El Niño‐Southern Oscillation (ENSO) and Southern Annular Mode (SAM), which affect temperature in the north, north/central and south of the continent, respectively (Flantua et al., 2016; see e.g. Box 1, Figure 1 IPCC AR5, 2013). With respect to topography, warming is generally projected to be stronger at higher than at lower elevations, with some aspect‐elevation combinations at mid‐to‐high elevations facing projected temperatures increases of up to 4°C for minimum temperature. This was the case for Cotahuasi, Titicaca‐Madidi, Uyuni, Sucre‐Potosí and Salta‐Jujuy, all located between 14°S and 24°S. There was no consistent difference in patterns of change between slope directions. The relationship between elevation and climate change is also complex, and processes related to albedo‐snow, cloud, water vapour and aerosol feedbacks contribute to an elevation‐dependent climate response (Mountain Research Initiative EDW Working Group, 2015), with generally greater warming trends at high elevations (Rangwala & Miller, 2012). However, there is not yet full agreement on this topic across the Andes (Pabón‐Caicedo et al., 2020).

Sound data on current climate and projected future changes are vital for accurate projections of species distributions. Two of the most commonly used datasets in biodiversity studies are ‘WorldClim’ (Fick & Hijmans, 2017; Hijmans et al., 2005) and ‘CHELSA’ (Karger et al., 2017). Both are available at a high resolution (30 arc seconds≈1 km). While WorldClim is primarily based on interpolated weather station data, augmented and/or corrected using other data sources, CHELSA is based on statistical downscaling of the ERA‐Interim reanalysis (Box 1). In the Andes, these data are limited by the low number of weather stations, meaning that the region's climatic complexity with its strong gradients and associated climate processes is likely to be poorly represented (Box 1). This limitation particularly affects precipitation. According to Karger et al. (2017), CHELSA performs better than WorldClim at representing orographic rainfall patterns in topographically complex areas; however, a known bias is that orographic precipitation may be overestimated on flat terrain, and as for WorldClim, uncertainty values are not provided. Consequently, freely available high‐resolution climate data for the Andes, though widely used, need to be interpreted with care as these often might not be suitable for the used purposes of modelling in topographic complex regions. Our analyses were based on outputs from Global Climate Models (GCMs). Both GCMs and Regional Climate Models (RCMs) provide ‘physically robust’ climate data, but, among other things, are limited by their coarse resolution (~140 km for GCMs and ~20–50 km for RCMs; Box 1).

BOX 1 **Climate data and climate models: Details on the climate datasets, the common approaches employed for their generation, and details about their reliability and uncertainties. Glossary of acronyms and specialised terms are available on‐line at https://www.ipcc‐data.org/guidelines/pages/glossary/glossary_b.html**

**Observational data**

Different types of climate data are used to produce climate information. The way in which each data type is obtained determines their characteristics and thus the correct interpretation and use. In‐situ observations (e.g. weather station data) represent a measured variable at one spatial point. Other data type is remotely sensed data, which are provided on a spatial–temporal grid. These usually have global coverage, where each grid cell represents an area of, for example, 5–100 km^2^ depending on the variable and instrument. In‐situ and remotely sensed data can also be assimilated into Global Climate Models (GCMs) to produce global gridded and physically consistent datasets (called ‘reanalysis’). Reanalyses are considered ‘quasi‐observational’ data and play an increasingly important role in applied studies, such as the identification of relationships between current climate and natural processes such as glacier fluctuations, river discharges, vegetation dynamics and ecosystem services (Li et al., 2020). They have also been used as surrogates of in‐situ observed climate in data‐sparse regions (Doblas‐Reyes et al., 2021), notably in mountain areas.

**Global and Regional Climate models**

GCMs are coupled mechanistic models, which simulate past, present and future climate and contribute to a better understanding of climate variability and change. These models simulate the key components of the climate—for example, the atmosphere, oceans, ice and land masses as well as the interactions between them. The results of a GCM are provided on a global spatiotemporal grid (at spatial resolutions well above 0.5° = ~55 km). Due to their distinct construction and resolution, different GCMs simulate unalike future regional responses to anthropogenic global warming. The uncertainties are exacerbated in mountain regions (Flato et al., 2013), where the spatial resolution of climate models is a limitation to adequately represent the height of peaks, valleys, and slopes, and the complex mountain atmosphere. Dynamic downscaling techniques such as Regional Climate Models (RCMs) overcome some of the GCMs uncertainties along the Andes (Falco et al., 2019; Urrutia & Vuille, 2009). Nevertheless, the current spatial resolution of both the GCMs and RCMs makes it challenging to use their products to represent ecological processes that occur at a much finer scale than ~55 km. An alternative or complementary to dynamic downscaling is the application of statistical downscaling, a technique that consists of using different statistical methods to generate regional projections. The performance of models and methods for producing information about regional climate change, particularly for mountainous regions, has recently been assessed by the Intergovernmental Panel on Climate Change (Doblas‐Reyes et al., 2021).

**Very high‐resolution climate data used in ecological sciences**

The very high‐resolution global climate datasets (~ 1 km) WorldClim (Hijmans et al., 2005), WorldClim2 (Fick & Hijmans, 2017) and CHELSA (Karger et al., 2017) are primarily based on interpolated weather station data, and these datasets are widely used for ecological research. Although they have not been rigorously tested along the Andes, in other regions of the world questions arose about their capacity to represent the climate in topographically complex regions (Bedia et al., 2013), and in areas where there is a low density of weather stations—both of which is the case in the Andes. More recent climatologies attempted to improve data accuracy by integrating additional information. For instance, while the WorldClim baseline climatology is based on weather station data only, CHELSA is based on quasi‐mechanistic statistical downscaling of an atmospheric reanalysis, in which satellite information was additionally included to correct biases, and the more recent WorldClim2 also includes satellite information. Karger et al. (2017) claim that CHELSA performs better than WorldClim for the prediction of the orographic precipitation patterns. A dense, high‐quality network of weather station data would be beneficial for resolving continued uncertainty over data quality and which dataset is best to use.

Future climate projections in these climate datasets are obtained from models used in the Coupled Model Intercomparison Project (https://www.wcrp‐climate.org/wgcm‐cmip). Climate change is computed as the difference between the GCMs output for the baseline climatology and for the targeted years (future period) in each grid point of the climate model (typical resolution ~200 km). These changes are interpolated to the high (~1 km) resolution grid and are added to the baseline climatology in high resolution (Fick & Hijmans, 2017). The assumption made is that the change in climate is stable over space (high spatial autocorrelation), a premise not achieved in regions with strong gradients as the Andean mountains as well as in other mountain ranges of the world (Maraun et al., 2017).

## 5 FUTURE VEGETATION RESPONSE PROJECTIONS

### 5.1 Biome level

**FIGURE 4:**
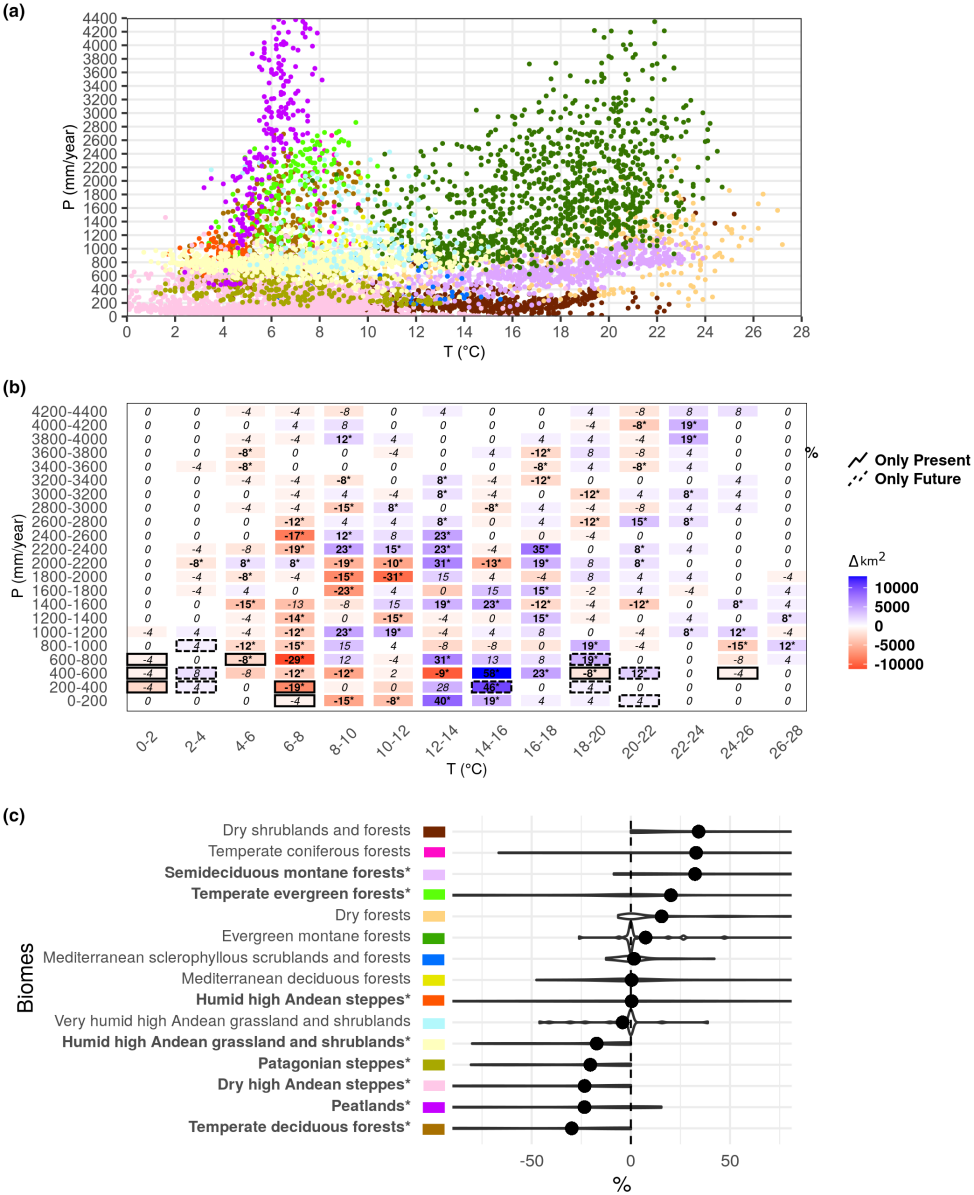
Projected changes in the climatic envelope area of Andean biomes. (a) Climatological classification of Andean biomes using annual mean temperature (°C) and total annual precipitation (mm) from CHELSA at 10‐min resolution (Karger et al. 2017) using bins measuring 2° temperature and 200 mm rainfall. Each point is a pixel of a given biome as indicated by the colour code displayed in the bottom panel. Axis y was truncated to 4400 mm (b) Projected change in the extent covered by specific combinations of annual mean temperature and total annual precipitation (bins measuring 2° and 200 mm rainfall). Changes are calculated as the difference between the future (2040–2070; RCP8.5 scenario) and near‐present conditions (1960–1990), using an ensemble of CMIP5 GCMs. Changes are expressed in absolute values (km^2^, coloured boxes) and relative values (% of change respect to 1960–1990, numbers inside boxes). Stars indicate levels of confidence (SNR at the 0.01 level). Full and dashed edge lines highlight combinations of temperature and precipitation found only in the historical and future scenarios, respectively. (c) Violin plots showing the projected relative change in the climatic envelope area for each biome using the climate classification shown in (a) in combination with the expected changes in (b). Each violin shows the distribution of the multi‐GCMs projected changes in the area covered by the present‐day climatic envelope of a biome (expressed in % of change relative to 1960–1990) where the dot is the ensemble mean. Stars indicate highly confident changes (SNR at the 0.01 level)

For each Andean biome, we first characterised their climatic niche (Figure 4a). From the 15 identified biomes, the broadest climate niche is found for the Evergreen montane forest and the narrowest for the Humid high Andean steppes in Patagonia. Dry shrublands and forest, Semideciduous montane forest, Dry forests and Evergreen montane forests all have mean temperatures above 12°C but differ in precipitation. Dry high Andean steppes and Humid high Andean grasslands and shrublands (locally known as dry Puna and humid Puna respectively) occupy mean temperatures between 0 and 12°C and receive total annual precipitation below 800 mm. Temperate deciduous and Temperate evergreen forests occur at temperatures between 4 and 10°C with an annual precipitation between 400–2200 and 800–2800 mm, respectively. Scattered peatlands exist along the Northern and Central Andes, but the Peatlands in southern Patagonia are a well identified vegetation unit spanning 400 km (Figure 1) with temperatures between 4 and 8°C and high levels of precipitation which are mostly above 1400 mm (Figure 4a).

The CMIP5 climate experiments project that areas with present‐day temperatures ranging from 6 to 8°C and with total annual precipitation lower than 1600 mm will decrease in extent (Figure 4b). In contrast, regions with present‐day temperatures of 14–18°C and total annual precipitation lower than 800 mm are projected to increase in extent. Some combinations of temperature and precipitation, for example 20–22°C and precipitation 400–600 mm, are not recorded at present but are projected to occur in the future (Figure 4b) while areas in the colder and drier extreme (<−2°C and below 800 mm) are projected to disappear. Given the coarse resolution of CMIP models, these values may slightly vary and should be used with caution.

Overlaying the projected climate changes with the current climatic niches highlighted differential impacts on the different biomes. The climatic envelope area of the Humid high Andean steppes might remain unchanged (though with high range of estimates), while for five biomes it will significantly likely decrease (Figure 4c), namely Temperate deciduous forest (−30%), Peatlands (−23%), Dry high Andean steppes (−23%), Patagonian steppes (−20.6%) and Humid high Andean grassland and shrublands (−17%). This decrease is in agreement with results from a previous study for the Dry high Andean steppes (Xeric puna) and the Humid high Andean grassland and shrublands (Humid puna; Tovar et al., 2013) but the magnitude of change in our results is higher. Another study by Ramirez‐Villegas et al. (2014) projected a decrease in species richness for the Humid Puna as a result of decrease in the available suitable climate area. For Temperate deciduous forest, Peatlands and Patagonian steppes, our study is the first to provide estimates of changed in climate envelope area under future climate conditions.

Our results also show that the climatic envelope area of Semideciduous montane forest (Selva Tucumano‐Boliviana) and Temperate evergreen forest (Bosque Valdiviano) will likely increase by 30% and 21%, respectively (Figure 4c). Previous studies have shown contrasting projections for the Semideciduous montane forest biome. On one hand, it is projected to have a substantial decrease in extent for the Argentinian side (Pacheco et al., 2010), in suitable area for key species (e.g. *Alnus acuminata*, Betulaceae; Wicaksono et al., 2017) and in plant species richness (Ramirez‐Villegas et al., 2014). On the other hand, stable areas under future climate change have been projected for the Bolivian side (Tovar et al., 2013). We did not find any previous studies projecting plant distributions for the Temperate evergreen forest.

Lastly, for seven biomes namely Dry shrublands and forests, Temperate coniferous forests, Dry forests, Evergreen montane forests, Mediterranean sclerophyllous scrublands and forests, Mediterranean deciduous forests and Very humid Andean grassland and shrublands, the uncertainty between climate model outputs is too large to reach a conclusion.

**FIGURE 5:**
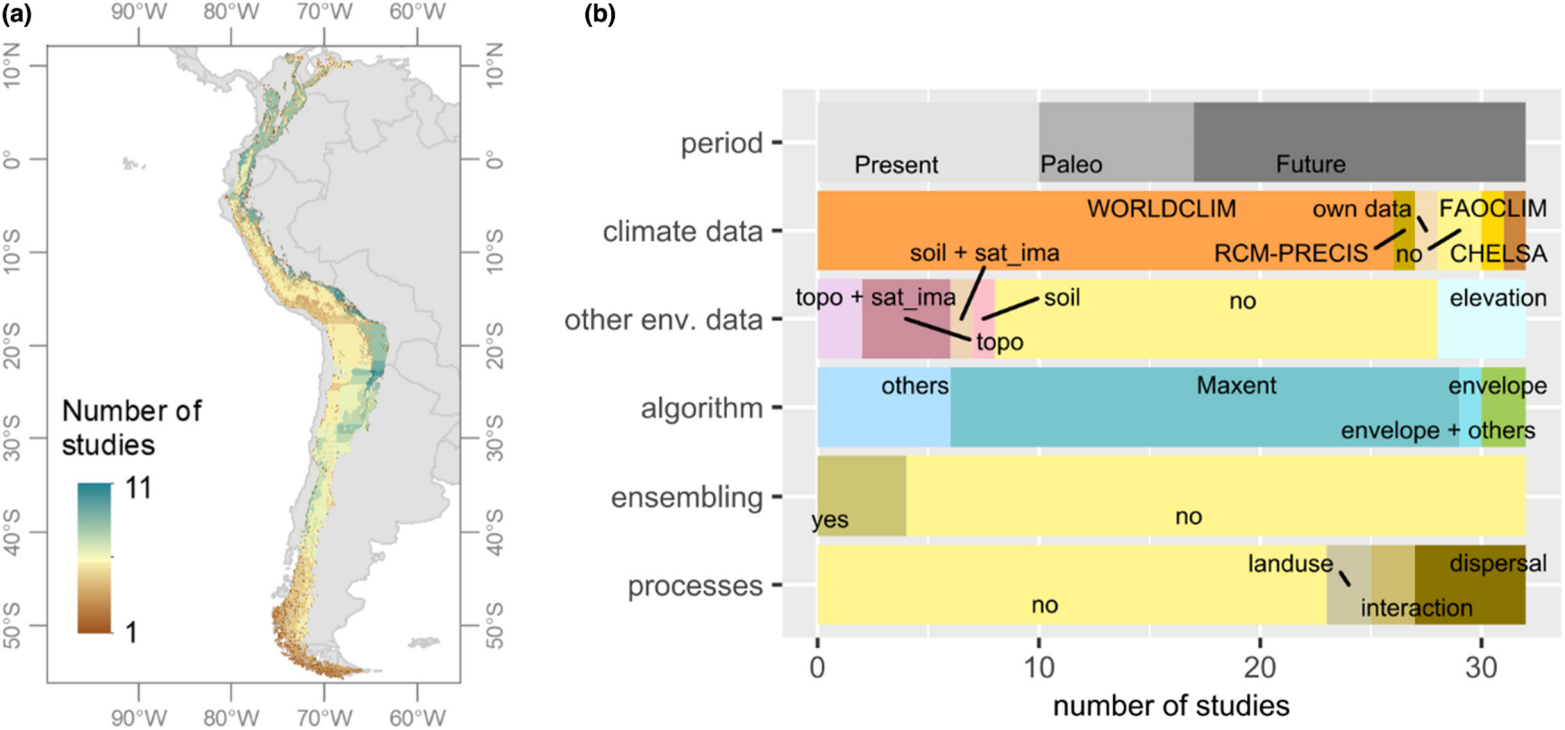
Summary of studies that have used species distribution models (SDMs) in Andean regions. (a) Geographical distribution of the 32 studies carried out in the Andes that used SDMs published between 2010 and 2019. (b) Model details used by these studies considering the studied period, the climatic data used, whether other environmental variables in addition to climatic data were used, the algorithm, whether model ensemble was applied or not, and whether other biological processes beyond climate were used. Topo = topographical, sat_ima = variables derived from satellite images. Details of the different studies are found in Table S2

Previous studies using distribution models to analyse responses to warming in the Andes have mostly focused on the Evergreen montane forests (Table S2; Figure 5a). Evergreen montane forests are already experiencing an upward migration, with mid‐elevation species suffering area loss (Feeley et al., 2011; Feeley & Silman, 2010). In addition, these forests have been projected to have a decrease in area and species richness (Ramirez‐Villegas et al., 2014; Tovar et al., 2013) and their tree species have been projected to have 15% increased risk of extinction (Tejedor Garavito et al., 2015). However, in our study, projections of change show a large spread between climate models for this biome. Differences in the projections could be attributed to the different modelling approaches (see next section).

Our results provide the first regional assessment of projected climate change impacts for the whole Andes (2040–2070). They are based on a worst‐case scenario in which radiative forcing (= net change in energy flux in the atmosphere) reaches 8.5 Watts per m^2^ by 2100, leading to a mean temperature increase over continental areas of around 5°C relative to pre‐industrial times (Collins et al., 2013). If pledges made at COP26 are held, then a current best‐case scenario would be a warming of 1.8°C, which would significantly reduce threats to thermally sensitive systems such as high Andean vegetation and temperature deciduous forests. However, although the magnitude of changes will vary according to the level of warming, the analyses here serve to highlight the biomes most sensitive to climatic change in the Andes. Future research should consider a combined modelling approach (rather than modelling individual biomes) and include a range of plausible scenarios, as well as an uncertainty analysis. In some cases, the large inter‐GCMs spread noted here was due to a few models performing as outliers. Future studies should identify the best subset of GCMs to be used for the Andes.

### 5.2 Species level: State of the art of distribution modelling of Andean plants

#### 5.2.1 Species Distribution Models (SDMs)

Most studies using SDMs have been conducted on species from the Evergreen montane forest biome and the Semideciduous montane forests, whereas species in the biomes of the Southern Andes are the least studied (Table S2; Figure 5a). Most of these studies have used only climatic data, without considering topographic and soil data (Figure 5b). For climate, the WorldClim dataset (Hijmans et al., 2005) was most widely used, while few studies have used alternative datasets such as Regional Climate Models used in the Mediterranean region of Chile (Bambach et al., 2013). The more recently published climate dataset, CHELSA (Karger et al., 2017), has yet to be used more widely in studies modelling Andean species. Important to note here is that a recent study shows little agreement between different climate datasets for the Andes (Morales‐Barbero & Vega‐Álvarez, 2019), that is, the choice of climate data will impact the model outputs.

Although there is a variety of algorithms with different levels of complexity for SDMs, only a limited number of algorithms are being applied (Table S2; Figure 5b). One of the simplest approaches is the ‘Envelope model’, where the species' niche is defined by the lower and upper bounds of environmental values at the locations where the species has been recorded (Guisan & Zimmermann, 2000). An example is the use of the species elevational range to model the present‐day distribution and potential changes under climate change scenarios of Andean trees (Feeley & Silman, 2010). While envelope models may be most intuitive, the most popular algorithm is MAXENT, a machine‐learning algorithm which iteratively matches the environment at the locations of projected occurrence to the environment at the actual occurrences while ensuring the solution has maximum entropy (i.e. probability distribution is closest to uniform) (Elith et al., 2011). Ensemble models, where two or more algorithms are combined (Hao et al., 2019), have been rarely used in Andean studies. Although the best practices of how to perform ensemble models have yet to be refined (Hao et al., 2019), they are useful to account for model uncertainty, especially in complex regions such as the Andes.

Biotic interactions (e.g. competition and mutualism) are rarely incorporated into SDMs in general, and studies focusing on the Andes are no exception. The few studies we identified for the Andes modelled interacting species separately and then assessed the spatial overlap of their distributions. For example, a study of the future projected distribution of 11 Argentinian cactus species and their pollinators found little mismatch between them under future warming (Gorostiague et al., 2018). Another study focusing on palaeodistributions of *Calceolaria* species in combination with their pollinators predicted floral traits divergence in Patagonia (Sosa‐Pivatto et al., 2017). Another key ecological process, dispersal, has been incorporated into modelling studies by only considering two extreme scenarios: full (unlimited) or null (restricted) dispersal (e.g. Bambach et al., 2013; Gorostiague et al., 2018; Ramirez‐Villegas et al., 2014). In an unlimited dispersal scenario, projections of future distributions use all suitable new areas, whereas in a null‐dispersal scenario, future distributions are only projected in areas where the species currently exists.

Among the main limitations of these models is the effect of niche truncation (when species occurrence records only represent a fraction of the climatic conditions the species could tolerate) on future projections (Peterson et al., 2018). Although improved sampling could help solve this issue, future conditions in the Andes might be non‐analogous to present‐day conditions (see Section 4) and thus palaeoecological data could be another valuable source of information (see Section 3). Lastly, our search did not identify any SDM studies for invasive species despite their potential negative impacts on biodiversity (but see Martin‐Gallego et al., 2020 on invasive species in temperate forests).

#### 5.2.2 Dynamic Vegetation Models (DVMs)

Dynamic vegetation models have emerged as an alternative to SDMs to simulate plant species ranges (Gutiérrez et al., 2016; Snell et al., 2014). DVMs include demographic processes and biotic interactions, which influence plant range dynamics, to project future vegetation composition and structure under climate change (Bugmann, 2001, 2014). For example, they explicitly include competition using parameters such as light interception of tree crowns and demographic rates such as plant growth, recruitment and mortality rates, and the influence of climate on these processes (Snell et al., 2014). Recent DVM development has also included dispersal to simulate species distributions (Snell, 2014). DVMs have successfully predicted range shifts driven by demographic processes in tree species (Bykova et al., 2012; Snell, 2014; Vanderwel et al., 2013). Despite the potential for DVMs to simulate plant ranges, one of their main limitations is the large number of parameters needed, which requires expert knowledge or empirical information on the ecology of species. DVM applications are often conducted in data‐rich regions (e.g. North America or Europe), and/or run for a limited set of well‐known or dominant species or plant functional types (Köhler & Huth, 1998; Rüger et al., 2020).

In the Andes, DVMs have been used to predict current vegetation composition and the structure of forest stands. For example, the first DVM modelled the dynamics of tree species in a low‐elevation tropical forest in the eastern Andes of Venezuela (Ramirez‐Angulo et al., 1997). Kammesheidt et al. (2001) studied the effect of different management strategies on forest composition using DVM, also in Venezuela. A similar study in south‐central Chile demonstrated how unsustainable logging impacted the composition of an old‐growth temperate rainforest (Rüger et al., 2007). In Bolivia, a DVM was used to simulate the ecotone between evergreen and deciduous forests (Seiler et al., 2014), and in Ecuador to study landslide impacts on forest structure in a tropical montane forest (Dislich et al., 2009). In south‐central Chile, DVMs have been applied to predict forest composition in several species‐rich forest stands near the Andes (Gutiérrez & Huth, 2012). The same DVM (FORMIND) was then applied to predict the influence of increased drought conditions driven by climate change on forest structure by 2100 (Gutiérrez et al., 2014).

The DVM applications discussed above have been conducted at local scales (e.g. forest stands <100 ha). To the best of our knowledge, there is no application of DVMs to study the dynamics of species distributions at a regional level for the Andes. However, there are examples for the use of DVMs at large spatial scales in South America to model changes in the forest carbon budgets of the Amazon (Brinck et al., 2017). Notwithstanding the promise of these techniques, challenges remain for the dynamic modelling of species ranges in the Andes, notably the paucity of data on dispersal and recruitment rates of individual plant species (Singer et al., 2016; a detailed discussion can be found in Snell et al., 2014).

## 6 RESEARCH PRIORITIES

To better understand Andean vegetation responses to climate change, several data and methodological gaps need to be filled. Below we summarise our view on the main research priorities. This is to enable the development of a coordinated research agenda to fill critical knowledge gaps (Table 1).

**TABLE 1:** Uncertainties and gaps in our understanding of past, present and future plant species distributions in the Andes and priorities for research

Topic	Subtopic	Uncertainties and gaps	Priorities
Observations	Species data	Number and list of native and non‐native species	Compile a plant species list for the whole Andes and per biome, including native and non‐native species
			Increase taxonomic treatments for Andean plant taxa
		Difference between under‐sampled and narrowly distributed species, spread of non‐native species	Increase species collections (native and non‐native) with high‐quality geographical and location data, beyond easily accessible areas
			Increase availability of existent specimen/occurrence data in public platforms Keep collecting to enable monitoring the spread of non‐native species and changes in native distributions
	Climate data	Observed trends and patterns of climate variability in specific regions and locations	Increase the collection of climate data at high frequency across the complete elevational gradient, significantly above the upper forest line
			Increase availability of existent climatic data, promoting a collaborative data‐sharing culture
			Increase the understanding of natural climate processes, including soil–vegetation–atmosphere interactions
		Spatial variation of temperature patterns at micro‐scales	Consider microclimatic variations using air and soil temperature sensors at finer spatial scales
Models	Climate models	The inability of models to represent clouds and convection	Develop new approaches to reduce errors directly related to shortcomings in process parametrisations
			Increase modelling resolution and complexity
		Poor land‐surface representation, including land surface–atmosphere interactions and feedbacks	Increase computational resources and technologies for archiving and sharing datasets
			Develop novel approaches to regional downscaling
	Plant distribution models	Representation of biological and ecological processes	Collect dispersal data and develop approaches to incorporate dispersal in SDMs and DVMs
			Collect demographic data (mortality, germination and establishment success) to improve parametrisation in DVMs and incorporate these data into SDMs
		Representation of external processes	Develop integrated models of land use change and plant distribution
			Include spatially explicit simulations of disturbance regimes (e.g. fire, building of infrastructure and roads)
		Model validation	Instal and monitor climate change experiments in field conditions Incorporate palaeo data in predictive models which could account for non‐analogous climate
		Representation of intraspecific variation	Collect data on functional traits on understudied areas, for natives and non‐native species, recording intraspecific variation (trait variation at population level), accounting for differences at local scales (phenotypic plasticity at elevational and latitudinal gradients, local adaptation)
		Spatial representation of DVM	Make DVMs spatially explicit, expanding their spatial scale without losing detail at local scales

### 6.1 Filling biological and climate data gaps

#### 6.1.1 Plant species data

A plant list for the whole of the Andes should be one of the first priorities. Although a recent list identifies around 28,700 species, this likely underestimates the real number of species as it is based solely on georeferenced records (Pérez‐Escobar et al., 2022). This list should contain information on estimated ranges and georeferenced locations of native and non‐native species, and should allow to estimate the number of species per biome. There is also a need to collect species occurrence data more widely, especially in remote areas and those with high endemism, including non‐native species that are spreading into the most remote ecosystems. In addition, many regional herbaria have yet to complete specimens' digitisation and currently key distributional records are only accessible through direct communication with them. Digitisation will allow participation in global (e.g. GBIF) or regional (e.g. SIB‐Colombia) initiatives of data storage and sharing.

#### 6.1.2 Invasive species

In the Andes, modelling non‐native plants, including those that are considered invasive species should be a priority given that many of them are spreading in the region, putting biodiversity and ecosystem services at high risk (Alexander et al., 2016; Pauchard et al., 2009). Examples include the invasion of non‐native conifers such as many *Pinus* species, alien shrubs like *Rosa rubiginosa* and herbs (Fuentes‐Lillo & Pauchard, 2019). Modelling the distribution of invasive species that are not at equilibrium is challenging, and recent studies have used co‐occurring native community members to improve predictions (Briscoe Runquist et al., 2021). Information about non‐native species' niches, associated species, demographic rates, dispersal capacity, residence time and on‐the‐ground microclimatic conditions is required for improved risk assessments of the spread and potential impact of such species.

#### 6.1.3 Climate data

A larger number of weather stations across the entire elevational gradient, aiming to collect information at high frequency, is needed to adequately capture climatic processes in the Andes and to increase the resolution along climatic gradients. Weather stations above the upper forest line in the Central Andes are scarce, limiting understanding of the climate in these vulnerable regions. At the same time, we should also aim to monitor microclimatic variations at local scales, such as initiated by the SoilTemp network using soil temperature sensors (Lembrechts et al., 2020).

As these data are collected, efforts should be made to share them widely. Either by developing regional platforms based on country efforts (e.g. BIOMODELOS, Colombia Velásquez‐Tibatá et al., 2019) or by contributing to existing global datasets (e.g. GBIF, BIEN, NOAA). Only a concerted effort between different research groups committed to generating and transferring knowledge will allow filling data gaps and mobilising data.

### 6.2 Advancing climate modelling

The advances in climate modelling are steps along a continuous revision and improvement process. Although state‐of‐the‐art GCMs simulate the first‐order statistics of large‐scale climatology appropriately, computational resources constrain GCMs to a simplified description of many physical processes, such as air flowing over the Andes range. Small‐scale details could be tracked using RCMs with increasing resolution/complexity as a higher spatial resolution is the priority for improved predictions of plant–climate interaction under future climate scenarios.

The global climate scientific community agrees that the largest uncertainties in climate models are associated with the representation of both cloud (sub‐grid) processes and land‐surface processes (including land cover and its management; Flato et al., 2013). Modelling the climate of the Andes is challenging because of the complexity of the processes and feedbacks to be simulated and because of the computational cost associated with the increased spatial resolution to appropriately represent its complex orography. Therefore, complementing the global simulations performed with GCMs with dynamic and statistical downscaling techniques should be the next step (see Box 1). However, this requires higher density and quality of observational data.

### 6.3 Improving plant distribution models

Detailed standards for distribution modelling have been recently published (e.g. Araújo et al., 2019). In the Andes, besides improving climate data and their resolution, other aspects deserve further attention in future studies such as incorporating key ecological, biological and palaeoecological knowledge into the models and their interpretations.

#### 6.3.1 Dispersal

One way to incorporate dispersal in SDMs, beyond using the extreme scenarios of null and full dispersal, is to use dispersal distances (e.g. migclim R package, Engler et al., 2012). This can be estimated using plant functional traits related to dispersal (Tamme et al., 2014). A similar approach can be followed for DVMs, coupling mechanistic seed dispersal models into plant regeneration modules (Snell, 2014). A less computationally and data‐intensive approach would be to group species by dispersal types and other functional traits and to model these entities instead (Tamme et al., 2014). However, only a handful of studies have collected dispersal traits in the Andes (e.g. Tovar et al., 2020), and further quantification of dispersal distances in the field is required.

#### 6.3.2 Demographic processes

There is a need to improve our understanding of how plant demographic processes, such as individual establishment and mortality, are being impacted by climate change. Long‐term vegetation monitoring using permanent plots is of particularly relevance to study demographic rates and to assess the influence of climate on them (Rüger et al., 2018). Current monitoring networks (e.g. RBA (https://redbosques.condesan.org/), MIREN (https://www.mountaininvasions.org/), GLORIA (https://redgloria.condesan.org/)) can help address this.

#### 6.3.3 Land use and fire regimes

Disturbances such as fire have been, and are, ubiquitous in the Andes. Representing disturbance dynamics such as fire regimes, logging, road construction and urbanisation, and other natural disturbances such as insect and pathogen outbreaks and landslides is thus necessary for a prediction of range dynamics. Incorporating disturbance and land‐use changes into plant distribution models adds important contextual information and is needed for both SDMs and DVMs.

#### 6.3.4 Spatial representation of DVMs

In the Andean temperate zone, where DVMs can be run at species level, future research can prioritise on how to expand the spatial scope without losing detail at local scales using an optimised species parameterisation procedure (Gutiérrez et al., 2016). Additional research should particularly focus on an improved definition of species niches, and factors affecting demographic processes, which shape the geographical distribution of species.

#### 6.3.5 Intraspecific adaptations, niches and traits

The distribution of species and biomes in the Andes has allowed the identification of species/biomes at risk and those showing higher resilience (e.g. Ramirez‐Villegas et al., 2014; Tovar et al., 2013). However, intraspecific variability in response to climate has been shown elsewhere, suggesting adaptive differences and response at the population level should be also analysed (Razgour et al., 2019). Given the large elevation gradient in the Andes, individual populations may show different levels of resilience to climate change through differentiated traits (such as thermal niches), and thus distributions should be projected separately for distinct populations. However, trait values are mostly obtained from only a few individuals, at species level or are averaged to obtain community‐trait‐weighted means, and thus trait data collection should consider intraspecific variation.

#### 6.3.6 Use of palaeoecological knowledge in SDMs

More cross‐disciplinary ground‐work should be done on integrating palaeoecological knowledge in predictive models—one of the spear points of the emerging discipline of Conservation Paleobiology (e.g. Dietl et al., 2015). This starts with considering palaeoecological knowledge during the stage of hypothesis development and validating outputs from species distributions models for past climate conditions with palaeoecological records (see more suggestions in supplementary material in Hooghiemstra & Flantua, 2019). In addition, fossil pollen data have shown that taxa can occupy different realised niches in the past, for example in the Andes, during the LGM, palaeo‐atmospheric pCO_2_ was different from present‐day values (Boom et al., 2002). Therefore, a series of recent papers warn against fitting only present‐day niches to reconstruct or predict species distributions for the past and future (e.g. Nogués‐Bravo, 2009; Veloz et al., 2012). SDMs can therefore be substantially improved if knowledge on past distributions is used for calibration and validation of the models.

### 6.4 Understanding species distributions across evolutionary time‐scales

#### 6.4.1 Changes in diversification rates on the Andes

Global climatic oscillations during the Pleistocene appeared to have influenced the speciation of endemic Andean plant groups in the Pleistocene onwards (Flantua et al., 2019), such as the genus *Espeletia* (Compositeae; Pouchon et al., 2018). A key research topic in the Andes is how diversification rates in lineages have changed across different evolutionary time‐scales in response to abiotic variables like climatic oscillations in the Andes. Modelling speciation and extinction rates as a function of time and paleo‐climatic variables provide a unique opportunity to understand how past climatic dynamics have affected the distribution of Andean plant lineages and the assemblies of their floras into discrete ecosystems (Condamine et al., 2013). More importantly, by integrating species occurrence data with speciation and extinction rates, it is also feasible to identify geographical areas that have the potential to serve as species pumps (i.e. areas with high speciation rates) or sinks (i.e. areas with high extinction rates; Forest et al., 2007; Pérez‐Escobar et al., 2017). The projection of speciation and extinction rates on geographical areas coupled with species distribution modelling supported by long fossil pollen records could enable the assessment of the survival of areas of importance for conservation because of their ‘evolutionary potential’ in the face of projected climatic conditions.

## 7 CONCLUSIONS

In this review, we have presented an analysis of projected climate change across the Andes and have summarised current existing information on climate change impacts on Andean vegetation.

First, we reviewed findings from Andean palaeoecological, dendrochronological and plot monitoring studies. We found that biome responses to climate change are and have been highly heterogeneous across the Andes. Main responses were, among others, (i) changes in elevational distributions of grassy/shrubs biomes, (ii) altered species composition due to upward shifts of warm‐adapted species or internal forest dynamics, (iii) primary succession in recently deglaciated areas, and (iv) changes in tree demographic patterns (recruitment and mortality) negatively affecting population viability.

Second, by exploring the projected climate changes for different regions across the Andes, we found that increasing temperatures are projected to be higher in the tropical Andes and at higher elevations (up to 4°C for 2040–2070, CMIP5 RCP8.5). Precipitation patterns are projected to be highly variable with clear differences between eastern and western slopes, yet, with large uncertainties in specific regions given the complex topography of the Andes. Climate change is likely to impact the distribution and extent of the Andean biomes. Projections for a worst‐case scenario (RCP 8.5) would result in a reduction of 17%–23% in the climate envelope area of the grassland/steppe biomes from the Central and Southern Andes, and 30% in that of Temperate deciduous forest, while the climatic envelope area for Temperate evergreen forest and Semideciduous montane forest may increase by 21% and 30%, respectively. Although current policy endeavours render this high‐forcing scenario as unlikely, it is useful to understand which biomes would be the winners, and which systems would be the losers and hence may deserve further attention, particularly given that there is still uncertainty around the sensitivity of the climate system. Most of the SDM studies we reviewed have been conducted in Montane evergreen forest while many vulnerable biomes such as the dry steppes of the Southern Andes remain understudied. Simultaneously, critical gaps in biological and climate data need to be covered. Lastly, mechanistic models, such as DVMs, have yet to be widely used in the Andes but offer a great potential especially for forested biomes.

Third, we identified four main research priorities: (i) Fill data gaps by working towards a comprehensive list of plant species for the Andean region, increasing species occurrence data, installing more weather stations above upper forest line and sharing data widely, (ii) advance climate modelling by representing key features in climate models (e.g. sharp rainfall gradients) and generating high‐resolution climate data with models that better represent the complex Andean topography, (iii) improve plant distribution models by including key ecological/biological processes, data on plant traits and available palaeoecological data and knowledge, but also by accounting for disturbance regimes and land‐use changes, and (iv) increase understanding of the locations and conditions which promote species diversification to support the integration of evolutionary refugia into conservation planning. Only a concerted effort between botanists, ecologists and climatologists working in the region will help achieve the proposed interdisciplinary research agenda.

## CONFLICT OF INTEREST

The authors have no conflict of interest to declare.

## BIOSKETCH

The authors are an interdisciplinary group including botanists, ecologists, geneticists, geographers and climatologists focusing on understanding plant dynamics and climate change in the Andes. This paper is one of the outputs of a Newton Fund Workshop organised in 2018 in Mendoza, Argentina. AA, AGG, AP, CT, DA, LF, OPE, PG and PF conceived the idea and outlined the paper, climate change analyses were designed by AAS, AFC, CGM, LF, PF, PZ, RCR and run by LF and PZ, the biome map was collated by AB, CT and JC. The writing was led by CT, AFC, AAG and SGAF. All authors wrote and/or contributed to the different sections of the manuscript based on their expertise.

## Supporting information


DataS1

## Data Availability

Data used in the climate diagnostics is publicly available from any of the open nodes of the 'Earth System Grid Federation' (ESGF) for example the node https://esgf.llnl.gov. Diagnostics used in the manuscript making use of this numerical climate data are described in detail in the methods. The results of the searches in the SCOPUS database are provided in the Supporting Information and the details of keywords and timeframe used for this are described in the methods.
