# Supplementary material for: Understanding climate change impacts on biome and plant distributions in the Andes: Challenges and opportunities
Source: J Biogeogr. 2022 Jun 3;49(8):1420–42. doi: 10.1111/jbi.14389 (PMC9543992; doi:10.1111/jbi.14389)
Supplement: Supplementary file 1 — DataS1 [file JBI-49-1420-s001.docx]

# Supplementary material

Methods S 1 Estimation of projected changes in Andean climate along slopes, differentiating areas based on their topography

To combine the models’ data in each selected 2°x2° bounding box, we took into account the mountain aspect and the elevation range, without interpolating the data from the individual models to a common grid, as described below:

Mountain aspect. We defined 3 categories: western slope, peak, and eastern slope, in latitudinal sections. So, for a given latitude, 'peak' corresponds to the grid cell with the maximum height, 'western slope' includes the grid cells west to the 'peak', and analogously, 'eastern slope' includes the grid cells that are east of the 'peak'.

Elevation range. We classified the Andes topography in vertical sections every 500 m, starting from 250 m up to 7000 m (which is the Aconcagua's height, the maximum peak of the Andes). Therefore, we got 11 elevation ranges [250 – 750 m), [750 – 1250 m), ..., [6750 – 7250 m).

For each grid cell of each CMIP5 model that fall within the 2°x2°lon/lat bounding box, we calculated its 'Mountain aspect' and 'Elevation range' working on its original grid.

Then, the data of each model were classified into the 33 different categories (combination of 3-aspect values and 11-elevation ranges).

For each 2°x2°lon/lat bounding box, we combined the set of CMIP5 models according to the 33 categories. Since the CMIP5 models have different resolutions, the 2°x2° bounding box will be covered by different combinations of grid cells. To combine them, a weighted average was performed taking into account the percentage of the grid area covered by each grid cell.

A signal-to-noise ratio (SNR) analysis was applied to the combined information of the 33 categories in each 2°x2° bounding box of climatic interest and tested for statistical significance by applying a student's t-test following the methodology of Kendon et al. (2008).

**References**

Kendon, E. J., Rowell, D. P., Jones, R. G., & Buonomo, E. (2008). Robustness of Future Changes in Local Precipitation Extremes. Journal of Climate, 21(17), 4280–4297. <https://doi.org/10.1175/2008JCLI2082.1>

Methods S 2 Evaluation of robustness of projected climate changes.

The robustness of the projected climate changes was assessed using the Signal to Noise Ratio (SNR) following Kendon et al. (2008). First the change in the mean signal is estimated as

${signal}_{i}=y_{i}-x_{i}$ (1)

where $y_{i}$ and $x_{i}$ are the mean area changes for each $i$ model for the future and present climate respectively. Using equation 1, the SNR is estimated as follows

$SNR=\frac{signal}{{\sqrt{\sigma_{x}^{2}+\sigma_{y}^{2}}}/2}$ (2)

Then, the test statistic is estimated by

$t=SNR\sqrt{\frac{n}{2}}$ (3)

and contrasted with its theoretical value for a t-Student distribution using α = 0.1 or 0.05. The degrees of freedom for a t-Student distribution are estimated as follows:

$$v=\left( n-1 \right)\left[ \frac{1+\left( \frac{\sigma_{y}}{\sigma_{x}} \right)^{2}}{1+\left( \frac{\sigma_{y}}{\sigma_{x}} \right)^{4}} \right]$$

**References**

Kendon, E. J., Rowell, D. P., Jones, R. G., & Buonomo, E. (2008). Robustness of Future Changes in Local Precipitation Extremes. Journal of Climate, 21(17), 4280–4297. <https://doi.org/10.1175/2008JCLI2082.1>

Fig. S 1 Number of pollen records collected in the Andes across the latitudinal gradient. The locations of available pollen records for Latin America were retrieved from an updated version (up to 2016) of Flantua et al. (2015) and intersected with the Andean biome map used in this study to generate the bars shown in the figure. In total we found 742 pollen records in the Andean region. Map shown in geographic coordinate system.


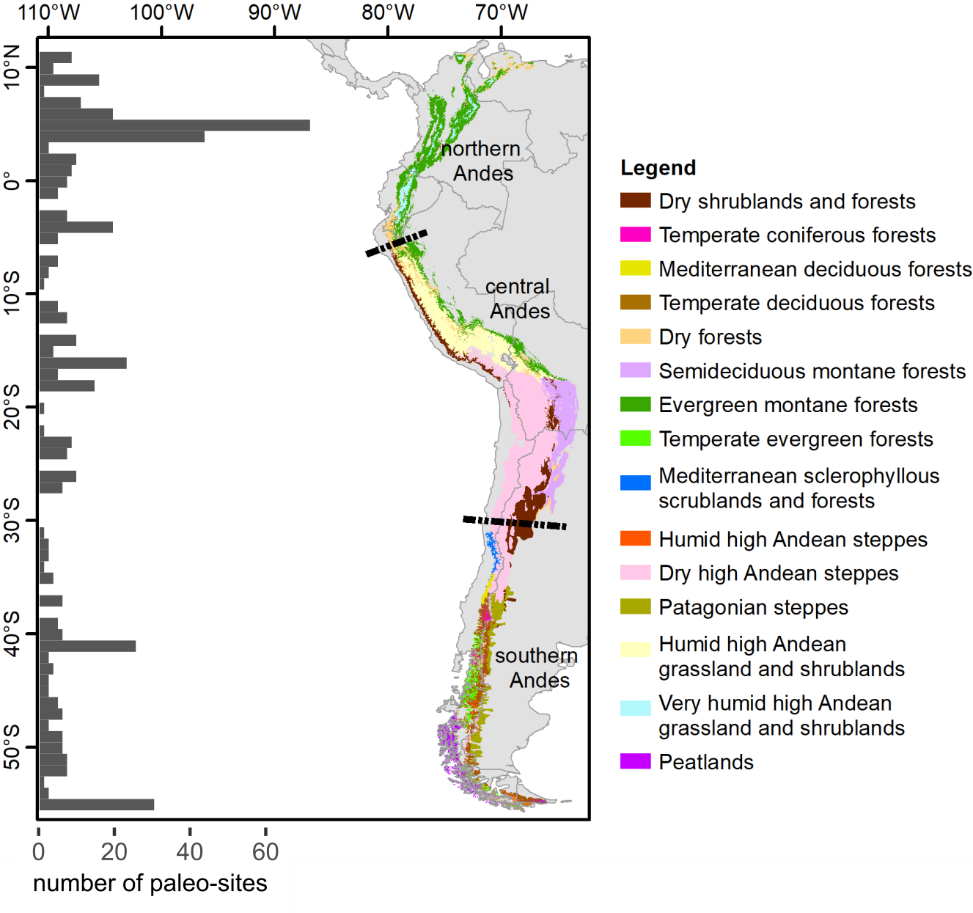


**References**

Flantua, S. G. A., Hooghiemstra, H., Grimm, E. C., Behling, H., Bush, M. B., González-Arango, C., Gosling, W. D., Ledru, M.-P., Lozano-García, S., Maldonado, A., Prieto, A. R., Rull, V., & Van Boxel, J. H. (2015). Updated site compilation of the Latin American Pollen Database. *Review of Palaeobotany and Palynology*, *223*, 104–115. <https://doi.org/10.1016/j.revpalbo.2015.09.008>

Fig. S 2 Climate change projections of annual-mean maximum near-surface air temperature (°C) in 19 locations along the Andes (see Fig. 1a), split by 500-meter elevation intervals (y-axis), and by aspect (western-slope, peak and eastern-slope in the x-axis). The changes are calculated using an ensemble of CMIP5 GCMs, as differences between the future (2040-2070; RCP8.5 scenario) and near-present (1960-1990) periods, without performing interpolations. Black edge lines highlight confident changes (SNR at 95%), while grey cells are combinations of elevation and slope without data.


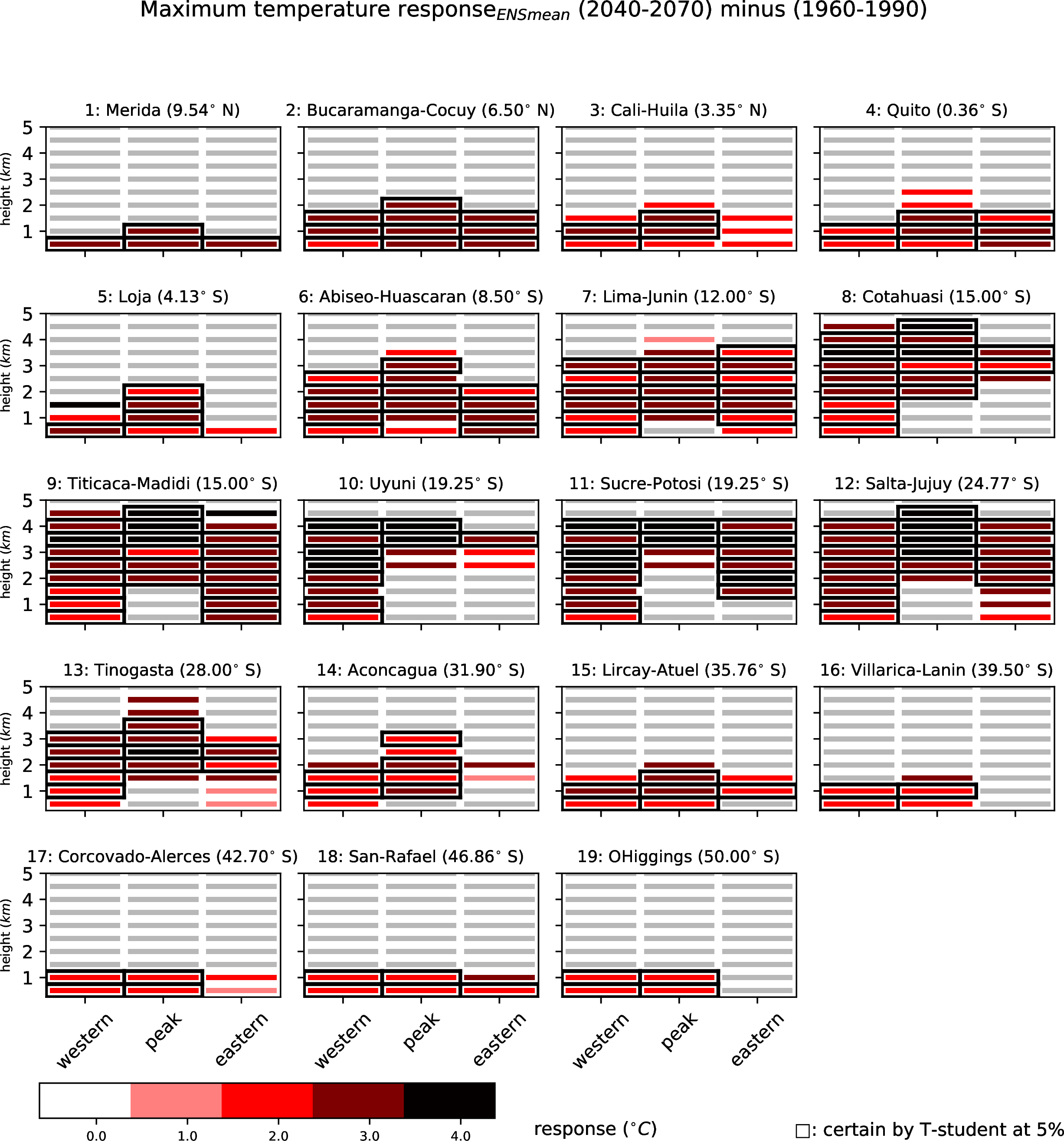


Table S 1 Studies that analyse medium- and short-term past climate changes (last 1000 years) and the vegetation responses in the Andes using dendrochronology, resurvey, chronosequences and plot monitoring. Studies were identified through a search in SCOPUS (see methods)

| Authors | Title | Country or region | Latitude | Longitude | Biome | Analysed period time | Recorded climate change | Methods to measure climate | Vegetation response | Methods to measure vegetation |
| --- | --- | --- | --- | --- | --- | --- | --- | --- | --- | --- |
| **Northern Andes** | | | | | | | | | | |
| Duque et al., 2015 | Thermophilization of adult and juvenile tree communities in the northern tropical Andes | Northwest Colombia |  |  | Evergreen montane forest | 2006-2014 | None | None | Mean rates of thermophilisation [thermal migration rate (TMR), °C·y−1] across all censuses were 0.011 °C·y−1 for adult trees and 0.027 °C·y−1 for juvenile trees. The observed changes in composition were driven primarily by patterns of tree mortality. | Permanent 1-ha forest inventory plots that were each censused at least twice each between 2006 and 2014. |
| Moret et al., 2019 | Humboldt’s Tableau Physique revisited | Ecuador |  |  | Very humid high Andean grassland and shrublands | 1802-2017 | Warming is assumed to have occurred in the last 200 years |  | A 215 to 266 m altitudinal shift of vegetation in the Antisana mountain over 215 years. | Historical records collected by Humboldt in 1802 and resurvey carried on in 2017 |
| Alvites et al., 2019 | Dendrochronological analysis and growth patterns of *Polylepis reticulata* (Rosaceae) in the Ecuadorian Andes | Ecuador | 2.55 S | 78.93 W | Very humid high Andean grassland and shrublands | 1800-2000 | No clear responses of tree growth to ENSO and vapor pressure deficit | Dendrochronology | None | None |
| **Northern and central Andes** | | | | | | | | | | |
| Báez et al., 2015 | Large-scale patterns of turnover and basal area change in Andean forests | Colombia, Ecuador, Perú, Argentina |  |  | Evergreen montane forests/Semideciduous montane forests | 1996-2009 | None | None | High elevation humid forests suffer from higher rates of tree mortality, possibly in response to increased temperature and lower precipitation rates. Lagged upslope tree migration. | Monitoring plots (Repeated censuses of trees ≥10 cm in 63 permanent plots) |
| Cuesta et al., 2019 | New land in the Neotropics: a review of biotic community, ecosystem, and landscape transformations in the face of climate and glacier change |  |  |  | Humid high Andean grassland and shrublands, Very humid high Andean grassland and shrublands | LGM to present | Deglaciation and warming | Literature review | Formation of new biotic communities in deglaciated areas, species upward shifts | Literature review |
| Fadrique et al., 2018 | Widespread but heterogeneous responses of Andean forests to climate change | Colombia, Ecuador, Peru, Bolivia, Argentina |  |  | Evergreen montane forest | 2000-2011 | Warming rate between 0 and 0.15 C/yr for most plots, very few plots had a negative warming rate, indicating decreased in temperature. Based on climate data 1990-2013) | CHELSA time series 1990-2013 | Thermophilisation, an increased in the composition of species with warmer thermal optima from lower elevations, is widespread across montane Andean forest. But rates of compositional change are not uniform across elevations. | Monitoring plots (n=200) sampled between 2000 and 2011 |
| **Central Andes** | | | | | | | | | | |
| Zimmer et al., 2018 | Time lag between glacial retreat and upward migration alters tropical alpine communities | Peru, Bolivia |  |  | Humid high Andean grassland and shrublands | 1975-2013 | Deglaciation since the 1970s due to warming | Glacier retreat analyses | Succession was affected by dispersal limitation due to overrepresentation of anemochorous species and poor facilitation process due to the infrequent presence of nurse plants | Four chronosequences |
| Morales et al., 2012 | Precipitation changes in the South American Altiplano since 1300 AD reconstructed by tree-rings | Peru, Bolivia, Chile |  |  | Dry highland steppes | 1300-2010 | Lower precipitation rates since 1970, droughts in the early years of the 1900. Driest years in the 700 yrs record are 1940-1982. | Dendrochronology | None | None |
| Ferrero et al., 2015 | Tree-ring based reconstruction of Río Bermejo streamflow in subtropical South America | Argentina | 23°13'S | 64°12'W | Semideciduous montane forests | 1680-2001 | Bermejo River streamflow reconstruction, sustained increase (related to positive trends in precipitation) since 1960. Also, higher occurrence frequency in extreme events in recent decades. | Dendrochronology | Sustained increase in tree growth (related to the increase in precipitation, thus streamflow) since 1960s | Tree rings of Juglans australis, *Cedrela lilloi* and *Schinopsis lorentzii* |
| Villalba et al., 1998 | Tree-ring evidence for long-term precipitation changes in subtropical South America | Argentina | 22° - 28°S | 64°40' - 65°53' W | Semideciduous montane forests | 1850-1980 | Increase in precipitation (and mean sea-level pressure) during last decades in subtropical Andes | Dendrochronology | Increasing tree radial growth due to increased precipitations | Tree rings of *Juglans australis* and *Cedrela lilloi* |
| Humanes‐Fuente et al., 2020 | Two centuries of hydroclimatic variability reconstructed from tree‐ring records over the Amazonian Andes of Peru | Peru | 10-12.5°S | 72.5-75°W | Evergreen montane forests | 1817-2010 | Drying trend from 2000-2009 , the driest in the 194-yrs reconstruction | Dendrochronology | Decrease in tree ring growth | Tree rings of *Juglans neotropica*, *Cedrela angustifolia* and *Cedrela odorata* |
| Ferrero et al., 2013 | Tree-growth responses across environmental gradients in subtropical Argentinean forests | Argentina | 22°19' - 27°10'S | 64°08' - 65°53'W | Semideciduous montane forests | 1890-1994 | None | Dendrochronology | Plant growth responses to climate along an elevation gradient: Positive responses of plant growth to precipitation at xeric and lower sites; positive responses of plant growth to temperature in higher and cooler sites; mixed responses at mid and milder sites | Tree rings of *Juglans australis*, *Cedrela lilloi* and *Schinopsis lorentzii* |
| Feeley et al., 2011 | Upslope migration of Andean trees | South‐eastern Peru | 13° 4'49.64"S [19S 221737] | 71°33'57.88"W [8552556] | Evergreen montane forests | 2003-2008 | None | None | Upward migration on trees occurring at genera level in present day: mean rate of migration is approximately 2.5–3.5 vertical metres upslope per year, and increasing abundances of tree genera previously distributed at lower elevations | Monitoring plots (Repeated censuses of trees (38 genera) ≥10 cm in 14 permanent plots) |
| Carilla et al., 2018 | Vegetation trends over eleven years on mountain summits in NW Argentina | Argentina | 26°40’ S | 65°44' W | Dry High Andean steppe | 2006-2017 | Increased climate variability | Instrumental (temperature data loggers) and global climate records (SOI) | Increase in species richness at 4000m and in species cover at 4700m, overall increase in diversity index in 11 years of monitoring, response to climate variability. Spatial patter: decrease in vegetation richness and cover with elevation (4000 to 4700 m asl) | Monitoring plots (64 - 1m2 high Andean plots in four summits from c. 4000 to c. 4800 m asl (GLORIA site), censused in 2006/08, 2012 and 2017) |
| **Central and southern Andes** | | | | | | | | | | |
| Garreaud et al., 2017 | The 2010-2015 megadrought in central Chile: Impacts on regional hydroclimate and vegetation | Chile | (two sampling sites, but extrapolated to central Chile 30-38°S) 32.4° S–70.5° W | 34.3° S–70.3° W | Mediterranean deciduous forests | 1000-2014 | Central Chile precipitation reconstruction. Dry conditions since the early 20th century. Precipitation during the last decade has been extraordinarily low in the context of the last millennium | Dendrochronology | Sustained decline in tree growth since the 20th century and more extreme in the last decade | Tree rings of *Austrocedrus chilensis* |
| Boninsegna et al., 2009 | Dendroclimatological reconstructions in South America: A review | South America | 5°-56°S | 62° - 73°W | Evergreen montane forests, Dry high Andean steppes, Dry shrublands and forests, Temperate coniferous forests, Temperate deciduous forests, Peatlands | 1640 - 2000 | Review showing different trends for temperature, rainfall, snow and regional atmospheric circulation | Literature review of dendroclimatic records and reconstructions studies in South America. Tree chronologies of over 11 species in different Andean biomes | Different species showed different climatic sensitive along the Andes, and even the same species showed differences in responses according to their location along environmental gradients. For example, *N. pumilio* sensitivity at tree line varied with latitude, from precipitation to temperature with increasing latitudes. Overall, trees growing at upper treeline are more sensitive to temperature variability | Literature review of studies where vegetation responses are measured through dendrochronology |
| **Southern Andes** | | | | | | | | | | |
| Holz & Veblen, 2012 | Wildfire activity in rainforests in western Patagonia linked to the Southern Annular Mode | Patagonia (Aysen) | 47-48° S | 73° W | Temperate evergreen forests | 1883-2002 | Increased in wildfire activity coincides with warming and drying trends during the 20th century and with positive phase of the Southern annular mode. | Tree-ring fires history from fire scars | None | None |
| Holz & Veblen, 2011 | Variability in the Southern Annular Mode determines wildfire activity in Patagonia | Patagonia (Aysen) | 47-48° S | 73° W | Temperate evergreen forests | 1500-2004 | Increased in wildfire activity coincides with the positive phase of the Southern annular mode. | Tree-ring fires history from fire scars | None | None |
| Mundo et al., 2017 | Fire history in southern Patagonia: Human and climate influences on fire activity in *Nothofagus pumilio* forests | Patagonia | 47-51° S | 72-73° W | Temperate deciduous forests | 1791-2010 | Low-frequency variations in fire recurrence are related to pulses of human population growth and settlement in southern Patagonia, whereas inter-annual variability in fire activity is strongly modulated by the Southern Annular Mode through its direct influence on regional | Dendrochronology | None | None |
| Veblen, 2010 | Wildfire Risk and Hazard in Northern Patagonia, Argentina | Northern Patagonia | 38-43° S | 71-72° W | Temperate deciduous forests | Last century | Fire risk is increasing due to warmer temperatures. Burning in 1998-1999 fire season coincided with driest calendar year and warmest spring in the last 90 year record | Literature review | None | None |
| Veblen et al., 1999 | Fire history in northern Patagonia: The roles of humans and climatic variation | Northern Patagonia | 39-43° S | 71-72° W | Temperate deciduous forests | 1500-1996 | Although climatic variability overrides human influences on fire regimes at an interannual scale, human activity is an equally important determinant of fire frequency at multidecadal scales. Years of extreme fire occurrence are associated with dry winter–springs of La Niña events and with the warm summers following El Niño events. | Dendrochronology | None | None |
| Villalba et al., 2003 | Large-scale temperature changes across the southern Andes: 20th-century variations in the context of the past 400 years | Patagonia | 37-55° S | 72-75° W | Temperate deciduous forests | 1640-2000 | The mean annual temperatures for the northern and southern sectors during the interval 1900–1990 are 0.53 ◦C and 0.86 ◦C above the 1640–1899 means, respectively. | Weather stations (1930-1990) and Dendrochronology (1640-2000) | None | None |
| Villalba, et al., 1998 | Tree-ring based reconstructions of northern Patagonia precipitation since AD 1600 | Northern Patagonia | 39-43° S | 70.5-71.5° W | Temperate deciduous forests | 1600-1988 | The twentieth century has the most variable and extreme intervals of drought and wetness since ad 1600. For the past 400 years, the three precipitation reconstructions show that the driest and wettest 25-year periods are 1895–1919 and 1925–1949, respectively. | Weather stations (1900-1990) and Dendrochronology (1600-1998) | None | None |
| Álvarez et al., 2015 | Relationships between climate variability and radial growth of *Nothofagus pumilio* near altitudinal treeline in the Andes of northern Patagonia, Chile | Chile | 39°53'S | 72°03'W | Patagonian steppes | 1930-2010 | Warmer growing seasons | Dendrochronology | Tree growth increased sharply from the 1960s to a peak in the early 1980s but subsequently declined for c. 30 years to its lowest level in >100 years. Declining rates of tree growth associated to warmer growing seasons. | Tree rings of *Nothofagus pumilio* |
| Kitzberger et al., 2001 | Inter-hemispheric synchrony of forest fires and the El Niño-Southern Oscillation |  |  |  | Patagonian steppes / South Western USA (Deserts) | 1650-1960 | Low fire frequency in 1780s–1830 coincides with decreased amplitude and/or frequency of ENSO | Tree-ring calibrated reconstructions of Southern Oscillation indices from several regional tree-ring networks, ice core databases, O18 time series recorded in corals | None | None |
| Kitzberger & Veblen, 1997 | Influences of humans and ENSO on fire history of *Austrocedrus chilensis* woodlands in northern Patagonia, Argentina | Argentina |  |  | Patagonian steppe | 1400-1900 | ENSO had a tentative effect on fire frequency. Periods of native human occupation increased fires. | Dendrochronology, fire scars | None | None |
| Mundo et al., 2012 | Multi-century tree-ring based reconstruction of the Neuquén River streamflow, northern Patagonia, Argentina | Argentina | 38°31'55"S | 69°24'49"W | Temperate deciduous forests / Patagonian steppes | 1346-2000 | Neuquen River streamflow reconstruction and extreme events. 20th century have some of the driest and wettest annual to decadal events in the past six centuries | Dendrochronology | None | None |
| Garibotti et al., 2011a | Spatiotemporal pattern of primary succession in relation to meso-topographic gradients on recently deglaciated terrains in the Patagonian Andes | Southwest Argentina | 50°17'S | 73°29'W | Patagonian steppe/ Humid high Andean steppes | 1699-1965 | Glacier retreat | Chronosequence with lichenometric dating | Species richness and cover increase in the younger and middle-age moraines, and then decrease in the older moraines. However, species richness and total cover is higher on the base than in the crest and mid-slope of moraine for each chronosequence. The successional sequence is characterized by a physiognomic development in fourth stage | 1 m^2^ sampling plots for terrestrial green algae, lichens, mosses, and vascular plants |
| Garibotti et al., 2011b | Vegetation development on deglaciated rock outcrops from glaciar Frías, Argentina | Central west Argentina | 41°10'S | 71°50'W | Patagonian steppe/ Humid high Andean steppes | 1868-1984 | Glacier retreat | Chronosequence with dates of the moraines, direct measurements of ice front positions, and the analysis of old terrestrial and aerial photographs | Successional change associated with increasing time since site deglaciation. Greater species richness and vegetation cover at older sites, but crustose lichens dominate younger sites. The vegetation assemblages were differentiated as the pioneer, mid-, or late-successional stage. | 1 m^2^ sampling plots for vascular plants and 50 m2 plots for cryptogamic flora |
| Speziale & Ezcurra, 2015 | Rock outcrops as potential biodiversity refugia under climate change in North Patagonia | Central west Argentina | 40°41' - 41°57' S | 70°33' - 71°22' W | Patagonian steppe/ Humid high Andean steppes | 2009 | Microclimatic differences (The south faces of the outcrops have lower maximum temperatures than the north faces and the matrix plots both in the spring and in the summer (approximately 5°C) | Temperature and humidity sensors and WorldClim database | At local scale, southern faces of outcrops had different species and lower maximum temperatures than insulated north faces and surrounding area | Sampling plots for plants, 10-30 m in diameter for outcrops and 100m^2^ in the vegetation matrix |
| Venegas-González et al., 2019 | Recent consequences of climate change have affected tree growth in distinct *Nothofagus macrocarpa* (DC.) FM Vaz & Rodr age classes in Central Chile | Central Chile | 32°57' - 34°51' S | 71°07' - 70°40' W | Mediterranean deciduous forests | 1980-2014 | Temperature increase and precipitation decrease since 1980 | Dendrochronology | Young trees are more sensitive to climate variability (less growth = less increase in basal area), however, seems that increasing of CO_2_ counteract these negative effect | Tree rings of *Nothofagus macrocarpa* |
| Venegas-González et al., 2018 | Effects of climate variability on growth and establishment patterns of *Nothofagus macrocarpa* in central Chile | Central Chile | 32°57' - 34°51' S | 71°07' - 70°40' W | Mediterranean deciduous forests | 1832-2014 | The last two centuries have presented 33 years of drought, and 9 decades in which de mean of the decade has high humidity. | Dendrochronology, instrumental data, Climatic Research Unit CRU TS3.24, KNMI Climate Explorer | During drought periods, there was less ring growth (at least> 64% of the years with low radial growth coincided with years of drought) and during humid periods more trees were established (between 14-41% of the trees were established in humid decades). | Tree rings of *Nothofagus macrocarpa*, 10-20 trees in each of the 10 populations in an area of 0.5-1 ha |
| Amoroso et al., 2015 | Does drought incite tree decline and death in *Austrocedrus chilensis* forests? | Argentina | 41°46'S | 71° 33'W | Temperate coniferous forest | 1914-2016 | Drought and climate variability (extreme events) | Tree cores and cross-sections processed and visually cross-dated. Regional precipitation and temperature records from nearby climate stations. | Radial growth decline and death of trees of *Astrocerdus chilensis* strongly associated with the occurrence of extreme drought events in late spring and summer. *A. chilensis* mortality is also explained by complex interactions between abiotic (e.g. site condition) and biotic factors (sex of trees & genetic variation) that contribute to stand-level decline. | Dendrochronology to determine i) radial growth decline of living and dead trees ii) tree mortality by radial growth patterns and canopy position. |
| Amoroso, Daniels, et al., 2012 | Temporal patterns of radial growth in declining *Austrocedrus chilensis* forests in Northern Patagonia: The use of tree-rings as an indicator of forest decline | Argentina | 41°46'S | 71° 33'W | Temperate coniferous forest | 1900-2000 | None | None | Radial growth decline of *A. chilensis*, with the length of the decline averaging 27 years and a maximum of 80 years. The decline preceded the external crown symptoms implying that water uptake has been reduced because of root damage from *Phyophthora* or drought or their interactions. | 0.1 hectare permanent plots at 12 sampling sites. All trees with DBH < 5 cm tagged. DBH, crown height class, health condition, tree cores. |
| Sangüesa-Barreda et al., 2019 | Detecting *Nothofagus pumilio* growth reductions induced by past spring frosts at the Northern Patagonian Andes | Argentina, Chile / North Patagonia | 35°-42°S |  | Temperate deciduous forest | 1900-1991 | Extreme climatic events in spring (warmer temperatures and late spring frosts) | Tree ring chronologies cross dated with climate stations close to sampling sites (Diguillin 1965-2016, Temuco 1965-2016, Bariloche 1939-2018). | Local growth reduction of *N. pumilio* was related to not only regional and local climate constraints but also caused by abnormal freezing temperatures in spring. Foliage damage of *N. pumilio* was related to abnormal warm springs followed by low minimum temperatures. Frost damages were greater in continental climates (Argentina). | Reconstruction of foliage damage occurrence by spring frosts in a deciduous forest (*Nothofagus pumilio*). Data from a tree ring network covering 30 *N. pumilio* stands surveyed in the field and 16 stands from the International Tree-Ring Data Bank. |
| Muñoz et al., 2014 | Patterns and drivers of *Araucaria araucana* forest growth along a biophysical gradient in the northern Patagonian Andes: Linking tree rings with satellite observations of soil moisture | Argentina, Chile, North Patagonia | 37.2°-40.2°S |  | Temperate coniferous forest, Patagonian steppes | 1750-1974 | Drought (soil moisture content) and warming | Tree rings related to regional climate (since 1750), and satellite observed surface soil and soil moisture variability (1979-2000) | Regional growth decline of *A. araucana* forests due to summer drying. Strong common regional signal in growth of *Araucaria* associated to moisture limitation during the growing season. Small differences on tree growth of *A. araucana* only along the longitudinal aridity gradient. It is expected a regional growth decline and increased tree mortality rates in these forests. | 21 tree-ring width chronologies from 37°S to 40°S of *A. araucana* to assess the spatio temporal patterns of tree growth |
| Castellano et al., 2019 | Climate-growth relationships of deciduous and evergreen *Nothofagus* species in Southern Patagonia, Argentina | Argentina y Chile | 45-53°S | 69-72° W | Temperate deciduous forest | 1850-2014 | Precipitation, temperature and SAM variability | Meteorological stations and SAM Index | A decrease in tree radial growth for both species (*N. betuloides* evergreen and *N. pumilio* deciduous) in the two most recent decades. Different climatic variable is related to a growth response for each species. If predicted warming 2-4 °C for the region occurs evergreen specie, growth for *N. betuloides* would be less. On the other hand, deciduous *N. pumilio* would maintain a higher growth rate due to favourable temperatures, as long as there are no marked drought periods. | Dendrochronology |
| El Mujtar et al., 2011 | Temporal progression trends of cypress mortality at permanent plots in a national forest reserve of *Austrocedrus chilensis* (Patagonia, Argentina) | Argentina | 41°S | 71°W | Temperate coniferous forests | 1988-2006 | None | None | Affected and dead trees increased with time in these plots, probably due to an outbreak of pathogens and low precipitation. | Permanent plots inventories |
| Fajardo et al., 2019 | Recent decadal drought reverts warming-triggered growth enhancement in contrasting climates in the southern Andes tree line | Chile | 36-54° S | NA | Temperate deciduous forest | 1950-2015 | Warming and drying trend after 1970s | Data from the Climate Research Unit (CRU) version 3.24 with gridded data at a 0.5º resolution | wet–cool conditions at high-elevation sites turned to be less frequent due to drier and warmer years during the late twentieth century. There is an aridity trend in the 20th century | Dendrochronology and tree ring wood isotopes |
| Holz et al., 2018 | Radial growth response to climate change along the latitudinal range of the world's southernmost conifer in southern South America | Chile | 39- 54° S | NA | Temperate evergreen forest/Peatlands | 1903-1993 | Warming after 1950s, and a drying trend for the period 1968-2001 in the Southern distribution of the focal tree species | Palmer Drought Severity Index [scPDSI]) data (1903–2006) for southern south America | *Pilgerodendron uviferum* radial growth changed after the 1950s, when warming arose in the region. Northern and southern edges showed a growth decline after 1976 and positive association to moisture. Instead at the species central distribution there was a negative relationship between tree growth and moisture (due to reduced radiation by clouds). | Dendrochronology |
| Mundo et al., 2010 | *Austrocedrus chilensis* growth decline in relation to drought events in northern Patagonia, Argentina | Argentina | 41° S | 71° W | Temperate coniferous forests | 1879-2006 | Extreme droughts in 1942 and 1943, cool and wet period in 1963 | Records from the Bariloche meteorological station combined with the 2.5° x 2.5°gridded Palmer drought severity index (Dai et al. 2004). | Warming decreased tree growth. Positive relationship between tree growth and precipitation. Severe droughts were recorded in the last 20 years in comparison with the previous 80 years. | Dendrochronology |
| Suarez et al., 2015 | Sensitivity of *Nothofagus dombeyi* tree growth to climate changes along a precipitation gradient in northern Patagonia, Argentina | Argentina | 41°07' 54'' to 41°23' 40''LS | 71°24'05'' to 71°48'46''LW | Temperate deciduous forests | 1476–2008 | Wet–cool conditions at high-elevation sites turned to be less frequent due to drier and warmer years during the late twentieth century. | Dendrochronology | The critical factor regulating tree growth is spring–summer water deficit induced by above-average temperature and reduced precipitation during the growing season. Regional increase in aridity at the end of twentieth century might have favoured the convergence to similar patterns in tree growth across the precipitation gradient in northern Patagonia. As wet–cool conditions at high-elevation sites turned to be less frequent due to drier and warmer years during the late twentieth century. | 12 tree-ring chronologies from *Nothofagus dombeyi*, at-mesic-to humid sites, to characterize climate–growth relationships along the west-to-east precipitation gradient. |
| Suarez et al., 2004 | Factors predisposing episodic drought-induced tree mortality in *Nothofagus* - Site, climatic sensitivity and growth trends | Argentina | 40°36 to 41°32' S | 71°16 to 71°40'W | Temperate deciduous forests | 1870-2000 | Decrease in precipitation with the extreme drought for 1998-1999 | Dendrochronology and instrumental records (meteorological stations 1905-1999) | Juveniles whose growth patterns showed sensitivity to drought were particularly likely to die. Older trees were less sensitive to climate. Trees with slower growth rate were more susceptible to drought. Susceptible trees may have been negatively affected by the drought that affected northern Patagonia in 1956–57. These results underscore the importance of considering drought-induced tree mortality as a non-random mechanism influenced by site, previous stress/disturbance history, ontogeny, vigour, climatic sensitivity and physiology. | Forests sampled of even aged, mixed *Nothofagus dombeyi* - *Austrocedrus chilensis* |
| Urrutia‐Jalabert et al., 2015 | Increased water use efficiency but contrasting tree growth patterns in *Fitzroya cupressoides* forests of southern Chile during recent decades | Chile | 40°10 - 41°32′S | 73°26′ - 72°35′ | Temperate evergreen forest and coastal region) | 300 years (Costal region), 1500 (Andean Cordillera) |  | Dendrochronology and Isotopes | Trees growing in the Coastal Range have become more efficient in their use of water, probably due to reduced stomatal conductance caused by increases in CO_2_ and warming. Trees growing in the Andes have also become more water use efficient, but this has been likely due to increased photosynthetic rates. The growth of forests under a more Mediterranean climate influence and restrictive soil conditions in the Coastal Range has been more negatively affected by current warming and drying; while the growth of old stands in the wet Andes has been positively affected by changes in climate (decreasing cloudiness) and increasing CO_2_. | Tree-ring growth and carbon isotopes of *Fitzroya cupressoides* in two stands growing in contrasting environmental conditions in the Coastal Range and Andean Cordilleras. In two 0.6 ha plots |
| Villalba et al., 2012 | Unusual Southern Hemisphere tree growth patterns induced by changes in the Southern Annular Mode | South America, Tazmania and New Zealand |  |  |  | 1400-2000 | Increases in Southern Annular Mode (SAM) (positive phase) in the las 4-5 decades reflected in drying and warming patterns | Dendrochronology | Patterns of tree growth between 1950 and 2000 differed significantly from those in the previous 250 years. Growth was lower in the dry-mesic forests of Patagonia. Variations in the Southern Annular Mode can explain 12–48% of the tree growth anomalies in the latter half of the twentieth century. | Tree-ring records from over 3000 trees in South America, Tasmania and New Zealand |
| Villalba & Veblen, 1997a | Spatial and temporal variation in *Austrocedrus* growth along the forest-steppe ecotone in northern Patagonia | Argentina | 34.04° a 43.11°LS | 70.36° a 71.42° LW | Temperate coniferous forests - Patagonian steppes ecotone | 1700-1989 | Lower precipitation in late spring-early summer or lower total annual precipitation, and higher temperatures were observed for several periods | Meteorological data averaged over 22 weather stations (~ 1904-1989) | Tree-ring growth decreased with lower seasonal (late spring-early summer) or annual precipitation, and with higher temperatures | 20 tree-ring chronologies of *Austrocedrus chilensis* |
| Amoroso, Suarez, et al., 2012 | *Nothofagus dombeyi* regeneration in declining *Austrocedrus chilensis* forests: Effects of overstory mortality and climatic events | Argentina/Northern Patagonia (Río Negro) | 41°46' S | 71°33' W | Temperate coniferous forests | 1900-2000 | Recent extreme droughts during last decades | Dendrochronology and instrumental records | Increased mortality of *Austrocedrus chilensis* by fungal decease ("mal del ciprés") may determine an increased regeneration of *Nothofagus dombeyi*, all related to drought events. Establishment of *N. dombeyi* under formerly pure *A. chilensis* forests near the eastern limit of *N. dombeyi* where precipitation is limiting, was recorded. This will probably lead to compositional and structural shifts. | Six *Austrocedrus chilensis* and *Nothofagus dombeyi* dominated stands were sampled by 1000 m^2^ plots. Living and dead trees were recorded. Trees were cored to reconstruct basal area increments and year of death. Tree regeneration was assessed by nine circular 30 m^2^ subplots by stand. |
| Camarero & Fajardo, 2017 | Poor acclimation to current drier climate of the long-lived tree species *Fitzroya cupressoides* in the temperate rainforest of southern Chile | Chile/Patagonia (Palena) | 42°22'55.2" to 42°23'16.8" S | 72°23'49.2" to 72°24'39.6" W | Evergreen Temperate coniferous forests and Temperate evergreen forests | 1840–2010 | Decrease in precipitation after 1970s | Isotope analyses, dendrochronology and instrumental records | *Fitzroya cupressoides* shows poor acclimation to recent drier conditions compared to *Nothofagus* species. *Nothofagus* appear to improve their use of water through changes in stomatal conductance. Results suggest that drying conditions can lead to decreasing trends in growth, regardless of temperature increasing trends, noticeable after 1980s | Three 1 ha-plots plots at different elevations, sampling 8-10 trees by each dominant species (*Nothofagus dombeyi*, *Nothofagus nitida*, *Saxegothaea conspicua* and *Fitzroya cupressoides*). and 2-3 stem cores per tree were extracted for growth rate and isotope analyses. |
| Christie et al., 2011 | Aridity changes in the Temperate-Mediterranean transition of the Andes since AD 1346 reconstructed from tree-rings | Chile (Maule to Biobío) and Argentina (Neuquén) | 35°52' to 38°03' S | 70°36' to 71°32' W | Mediterranean sclerophyllous scrublands and forests, in transition to Temperate coniferous forests | 1346-2004 | Occurrence rate of severe and extreme droughts steadily increased between XVI-XVIII centuries, remained constant during XIX century and significantly increased during the XX century. | Dendrochronology and modelled records that allowed 657 years-climate series reconstruction, Palmer Drought Severity Index (PDSI) | *Austrocedrus chilensis* population severely affected with less recruitment and higher mortality rates due to an increase in extreme drought events. | Increment cores from living trees and cross sections from dead trees and sub-fossil wood from open *Austrocedrus chilensis* stands in six study sites, totaling 650 tree-ring series.. |
| Kitzberger et al., 1997 | Climatic influences on fire regimes along a rain forest-to-xeric woodland gradient in northern Patagonia, Argentina | Argentina/Northern Patagonia | 40°38' S to 41°40' S | 71° to 71°50' W | Temperate evergreen forests in transition to Temperate coniferous forests | 1722-1990 | None, but identified fire frequency as a result of extreme droughts | Dendrochronology related to instrumental records and fire records | Annual variation in fire frequency and extent is strongly influenced by annual climatic variation (besides human activities). Fire in *Nothofagus* rainforests is highly dependent on drought during the spring and summer of the same year and less strongly favoured by drought during spring of the previous year. Fuel availability is dependent on the vegetation structure, occurrence of *Chusquea* bamboos flowering and intense drought occurrences. In contrast, fire in the xeric *Austrocedrus* woodlands is not simply dependent on drought but favoured by greater climatic variability over several years. | Fire chronologies derived from fire scars on trees (*Austrocedrus chilensis*, *Nothofagus dombeyi*, *N. antarctica*, *Fitzroya cupressoides* and *Pilgerodendron uviferum*) collected in 10 areas and related to tree-ring proxy records of climate over the period 1820-1974. Fire records for the period 1940-1988 were compared to instrumental weather data. |
| Lara et al., 2005 | Spatial and temporal variation in *Nothofagus pumilio* growth at tree line along its latitudinal range (35°40'-55° S) in the Chilean Andes. | Chile, from south-central Andes to Southern Patagonia and Argentina, Southern Patagonia | 35°36' to 54°57' S | 67°30' to 72°54' W | Temperate deciduous forest (mainly) and Mediterranean deciduous forests (marginally). | 1720-1999 | Decrease of temperatures from mid-1940 to mid-1970 dominated by summer warming, followed by a prominent increase after 1976 to 1999. Decrease in precipitation from 1936 to 1999. | Dendrochronology related to instrumental records | *Nothofagus pumilio* tree radial growth at the northernmost portion of the gradient showed a positive relationship with the highly seasonal-lower precipitation and negative with the higher temperature with high seasonality. Conversely, tree growth in most of the southern part of the gradient was positive correlated to higher mean annual temperature and little seasonality in precipitation. Temporal patterns of *N. pumilio* radial growth increased after 1963 as a response to a rise in mean annual temperature and summer warming, being more intense towards southern Patagonia but not reflected in a tree line upward movement. A decrease in precipitation during the period 1932 to 1986 for the southern-most part of the gradient was also shown. | Wood core samples were taken from 48 *Nothofagus pumilio* tree line sites, two cores from every tree. A composite tree-ring chronology was developed for each region, showing growth patterns in correlation with temperature and precipitation variation. |
| Arco Molina et al., 2019 | Variations in the intrinsic water-use efficiency of North Patagonian forests under a present climate change scenario: Tree age, site conditions and long-term environmental effects | Argentina/Northern Patagonia (Neuquén) | 37°49'48'' to 39°13'12" S | 70°34'12" to 71°15'36" W | Temperate coniferous forests | 1277-2013 | Increased mean annual maximum temperature during the last century, noticeable during active plant growth period (November to January). | Isotope analyses, dendrochronology and modelled records (CRU) | *Araucaria* showed an increased intrinsic water-use efficiency during last century due to climate change and atmospheric CO2 but responses in growth rates were not related because of drought stress or soil nutrient limitation, showing in xeric sites the highest basal area increment compared to those in mesic ones. | Four *Araucaria araucana* forests were sampled from mesic and from xeric conditions. Four trees were selected per age class and per site (32 trees in total). Three to four cores were taken from each tree for isotope analysis (Delta 13C ratio and intrinsic water-use efficiency) |
| Villalba & Veblen, 1998 | Influences of large-scale climatic variability on episodic tree mortality in northern Patagonia | Argentina/Northern Patagonia | 39 to 43 ° | 71°06' to 71°42'' | Temperate coniferous forests | 1906-1989 | Warm-dry and cool-wet periods | Dendrochronology related to instrumental records | Mortality of *Autrocedrus chilensis* related to extreme droughts and ENSO events (warm and dry springs and summers). Establishment of *A. chilensis* related to cool-wet periods | Cross sections from dead trees in 9 stands at 6 sites near the forest-steppe ecotone |
| Villalba et al., 1997 | Recent trends in tree-ring records from high elevation sites in the Andes of northern Patagonia | Argentina/Northern Patagonia | 41°05 to 41°20 | 71°36 to 71°54 | Temperate deciduous forest | 1750-1990 | Warmer conditions started in the summer of 1977–1978, persisting during the 1980s. Increased length of the snow-free period is observed during the last 50 years. This mean annual temperatures are not unprecedented in the last 250 years | Dendrochronology and instrumental records (temperature, precipitation and snow cover index | Tree growth responses of *Nothofagus pumilio* to climatic variations at exposed high-elevation sites where snow accumulation is limited by the strong winds, tree-growth appears to be more related to temperature fluctuations. In contrast, at sites where the snow cover persists for longer times, tree growth is more sensitive to snow persistence | Dendrochronology, three transects along a precipitation gradient (8 in the wettest zone, 4 in the mesic site and 2 in the dry zone) |
| Rodríguez-Catón et al., 2019 | Radial growth patterns associated with tree mortality in *Nothofagus pumilio* forest | Argentina/Northern Patagonia | 40°35'32.6''/41°15'41.9''/41°46'41.0'' | 71°08'33.0''/71°16'57.4''/71°35'21.0'' | Temperate deciduous forest | 1860-2015 | Extreme drought in 1978-1979 | Literature | *Nothofagus pumilio* mortality and population declined after 1978 | Chronologies of basal area increment in live and dead trees at three sites in northern Patagonia. Calculation of the probability of death with logistic models. |
| Villalba & Veblen, 1997b | Regional patterns of tree population age structures in northern Patagonia: Climatic and disturbance influences | Argentina/Northern Patagonia | 39°17' to 42°59' | 71° to 71°29' | Temperate coniferous forests | 1700-1989 | Warm and dry conditions alternate with cool and wet conditions, however, throughout the 1980s, with the exception of the summer of 1984, a warm-dry pattern prevails | Instrumental data (1906-1989) related to tree-ring records | Almost no *Austrocedrus* establishment has occurred since the warm-dry period began in c. 1980. In contrast, many trees established during the cool-wet conditions between 1963 and 1979 with peaks in numbers of surviving trees corresponding to particularly cool-wet conditions in 1964-66 and 1973-75 | On 26 plots of varying size, establishment data were recorded, and ring width chronologies were performed. |
| Villalba, 1994 | Tree-ring and glacial evidence for the medieval warm epoch and the little ice age in southern South America | Central Chile/ Argentina Southern and Northern Patagonia | 32°38'/41°11'/48° to 51° | 70°45/71°44' | Temperate coniferous forests | ~800-2000 | Intervals of high recurrence of the Southern Oscillation (SO) events alternate with low event recurrence. During the 13th century (concurrent with the Medieval Warm Period), precipitation in central Chile and summer temperature in northern Patagonia were above average. Warm-type events of the SO predominated over cold-ones during the Medieval. The prevalence of negative departures of winter rainfall and summer temperature from A.D. 1280 to 1380, and from A.D. 1520 to 1650, simultaneous with the Little Ice Age, could be related to a predominance of cold- over warm-type events of the SO. | Glacier records and tree-ring reconstructions | None | None |
| von Gunten et al., 2009 | A quantitative high-resolution summer temperature reconstruction based on sedimentary pigments from Laguna Aculeo, central Chile, back to AD 850 | Central Chile | 33°50' | 70°54' | Mediterranean sclerophyllous scrublands and forests | 850-2000 | Medieval Climate anomaly between 1150 and 1350. Little Ice Age starting 1380 and ending 1750-1880 | Summer temperature reconstruction from sediment core from the lagoon and age model | None | None |

**References**

Álvarez, C., Veblen, T. T., Christie, D. A., & González-Reyes, Á. (2015). Relationships between climate variability and radial growth of Nothofagus pumilio near altitudinal treeline in the Andes of northern Patagonia, Chile. *Forest Ecology and Management*, *342*, 112–121. https://doi.org/10.1016/j.foreco.2015.01.018

Alvites, C., Battipaglia, G., Santopuoli, G., Hampel, H., Vázquez, R. F., Matteucci, G., & Tognetti, R. (2019). Dendrochronological analysis and growth patterns of Polylepis reticulata (Rosaceae) in the Ecuadorian Andes. *IAWA Journal*, *40*(2), 331-S5. https://doi.org/10.1163/22941932-40190240

Amoroso, M. M., Daniels, L. D., & Larson, B. C. (2012). Temporal patterns of radial growth in declining Austrocedrus chilensis forests in Northern Patagonia: The use of tree-rings as an indicator of forest decline. *Forest Ecology and Management*, *265*, 62–70. https://doi.org/10.1016/j.foreco.2011.10.021

Amoroso, M. M., Daniels, L. D., Villalba, R., & Cherubini, P. (2015). Does drought incite tree decline and death in Austrocedrus chilensis forests? *Journal of Vegetation Science*, *26*(6), 1171–1183. https://doi.org/10.1111/jvs.12320

Amoroso, M. M., Suarez, M. L., & Daniels, L. D. (2012). Nothofagus dombeyi regeneration in declining Austrocedrus chilensis forests: Effects of overstory mortality and climatic events. *Dendrochronologia*, *30*(2), 105–112. https://doi.org/10.1016/j.dendro.2010.12.005

Arco Molina, J. G., Helle, G., Hadad, M. A., & Roig, F. A. (2019). Variations in the intrinsic water-use efficiency of north Patagonian forests under a present climate change scenario: Tree age, site conditions and long-term environmental effects. *Tree Physiology*, *39*(4), 661–678. https://doi.org/10.1093/treephys/tpy144

Báez, S., Malizia, A., Carilla, J., Blundo, C., Aguilar, M., Aguirre, N., Aquirre, Z., Álvarez, E., Cuesta, F., Duque, Á., Farfán-Ríos, W., García-Cabrera, K., Grau, R., Homeier, J., Linares-Palomino, R., Malizia, L. R., Cruz, O. M., Osinaga, O., Phillips, O. L., … Feeley, K. J. (2015). Large-Scale patterns of turnover and basal area change in Andean forests. *PLOS ONE*, *10*(5), e0126594. https://doi.org/10.1371/journal.pone.0126594

Boninsegna, J. A., Argollo, J., Aravena, J. C., Barichivich, J., Christie, D., Ferrero, M. E., Lara, A., Le Quesne, C., Luckman, B. H., Masiokas, M., Morales, M., Oliveira, J. M., Roig, F., Srur, A., & Villalba, R. (2009). Dendroclimatological reconstructions in South America: A review. *Palaeogeography, Palaeoclimatology, Palaeoecology*, *281*(3–4), 210–228. https://doi.org/10.1016/j.palaeo.2009.07.020

Camarero, J. J., & Fajardo, A. (2017). Poor acclimation to current drier climate of the long-lived tree species Fitzroya cupressoides in the temperate rainforest of southern Chile. *Agricultural and Forest Meteorology*, *239*, 141–150. https://doi.org/10.1016/j.agrformet.2017.03.003

Carilla, J., Halloy, S., Cuello, S., Grau, A., Malizia, A., & Cuesta, F. (2018). Vegetation trends over eleven years on mountain summits in NW Argentina. *Ecology and Evolution*, *8*(23), 11554–11567. https://doi.org/10.1002/ece3.4602

Castellano, P. L., Srur, A. M., & Bianchi, L. O. (2019). Climate-growth relationships of deciduous and evergreen Nothofagus species in southern Patagonia, Argentina. *Dendrochronologia*, *58*, 125646. https://doi.org/10.1016/j.dendro.2019.125646

Christie, D. A., Boninsegna, J. A., Cleaveland, M. K., Lara, A., Le Quesne, C., Morales, M. S., Mudelsee, M., Stahle, D. W., & Villalba, R. (2011). Aridity changes in the Temperate-Mediterranean transition of the Andes since ad 1346 reconstructed from tree-rings. *Climate Dynamics*, *36*(7–8), 1505–1521. https://doi.org/10.1007/s00382-009-0723-4

Cuesta, F., Llambí, L. D., Huggel, C., Drenkhan, F., Gosling, W. D., Muriel, P., Jaramillo, R., & Tovar, C. (2019). New land in the Neotropics: A review of biotic community, ecosystem, and landscape transformations in the face of climate and glacier change. *Regional Environmental Change*, *19*(6), 1623–1642. https://doi.org/10.1007/s10113-019-01499-3

Duque, A., Stevenson, P. R., & Feeley, K. J. (2015). Thermophilization of adult and juvenile tree communities in the northern tropical Andes. *Proceedings of the National Academy of Sciences*, *112*(34), 10744–10749. https://doi.org/10.1073/pnas.1506570112

El Mujtar, V. A., Andenmatten, E., Perdomo, M., Letourneau, F., Grau, O., & Gallo, L. (2011). Temporal progression trends of cypress mortality at permanent plots in a National forest reserve of Austrocedrus chilensis (Patagonia, Argentina). *Forest Systems*, *20*(2), 209. https://doi.org/10.5424/fs/2011202-8916

Fadrique, B., Báez, S., Duque, Á., Malizia, A., Blundo, C., Carilla, J., Osinaga-Acosta, O., Malizia, L., Silman, M., Farfán-Ríos, W., Malhi, Y., Young, K. R., Cuesta C., F., Homeier, J., Peralvo, M., Pinto, E., Jadan, O., Aguirre, N., Aguirre, Z., & Feeley, K. J. (2018). Widespread but heterogeneous responses of Andean forests to climate change. *Nature*, *564*(7735), 207–212. https://doi.org/10.1038/s41586-018-0715-9

Fajardo, A., Gazol, A., Mayr, C., & Camarero, J. J. (2019). Recent decadal drought reverts warming‐triggered growth enhancement in contrasting climates in the southern Andes tree line. *Journal of Biogeography*, jbi.13580. https://doi.org/10.1111/jbi.13580

Feeley, K. J., Silman, M. R., Bush, M. B., Farfan, W., Garcia Cabrera, K., Malhi, Y., Meir, P., Salinas Revilla, N., Raurau Quisiyupanqui, M. N., & Saatchi, S. (2011). Upslope migration of Andean trees. *Journal of Biogeography*, *38*, 783–791.

Ferrero, M. E., Villalba, R., De Membiela, M., Ferri Hidalgo, L., & Luckman, B. H. (2015). Tree-ring based reconstruction of Río Bermejo streamflow in subtropical South America. *Journal of Hydrology*, *525*, 572–584. https://doi.org/10.1016/j.jhydrol.2015.04.004

Ferrero, M. E., Villalba, R., De Membiela, M., Ripalta, A., Delgado, S., & Paolini, L. (2013). Tree-growth responses across environmental gradients in subtropical Argentinean forests. *Plant Ecology*, *214*(11), 1321–1334. https://doi.org/10.1007/s11258-013-0254-2

Garibotti, I. A., Pissolito, C. I., & Villalba, R. (2011a). Spatiotemporal pattern of primary succession in relation to meso-topographic gradients on recently deglaciated terrains in the Patagonian Andes. *Artic and Alpine Research*, *43*(4), 555–567.

Garibotti, I. A., Pissolito, C. I., & Villalba, R. (2011b). Vegetation development on deglaciated rock outcrops from glaciar Frías, Argentina. *Arctic, Antarctic, and Alpine Research*, *43*(1), 35–45. https://doi.org/10.1657/1938-4246-43.1.35

Garreaud, R. D., Alvarez-Garreton, C., Barichivich, J., Boisier, J. P., Christie, D., Galleguillos, M., LeQuesne, C., McPhee, J., & Zambrano-Bigiarini, M. (2017). The 2010–2015 megadrought in central Chile: Impacts on regional hydroclimate and vegetation. *Hydrology and Earth System Sciences*, *21*(12), 6307–6327. https://doi.org/10.5194/hess-21-6307-2017

Holz, A., Hart, S. J., Williamson, G. J., Veblen, T. T., & Aravena, J. C. (2018). Radial growth response to climate change along the latitudinal range of the world’s southernmost conifer in southern South America. *Journal of Biogeography*, *45*(5), 1140–1152. https://doi.org/10.1111/jbi.13199

Holz, A., & Veblen, T. T. (2011). Variability in the Southern Annular Mode determines wildfire activity in Patagonia. *Geophysical Research Letters*, *38*(14), n/a-n/a. https://doi.org/10.1029/2011GL047674

Holz, A., & Veblen, T. T. (2012). Wildfire activity in rainforests in western Patagonia linked to the Southern Annular Mode. *International Journal of Wildland Fire*, *21*(2), 114. https://doi.org/10.1071/WF10121

Humanes‐Fuente, V., Ferrero, M. E., Muñoz, A. A., González‐Reyes, Á., Requena‐Rojas, E. J., Barichivich, J., Inga, J. G., & Layme‐Huaman, E. T. (2020). Two centuries of hydroclimatic variability reconstructed from tree‐ring records over the Amazonian Andes of Peru. *Journal of Geophysical Research: Atmospheres*, *125*(18). https://doi.org/10.1029/2020JD032565

Kitzberger, T., Swetnam, T. W., & Veblen, T. T. (2001). Inter-hemispheric synchrony of forest fires and the El Nino-Southern oscillation. *Global Ecology and Biogeography*, *10*(3), 315–326.

Kitzberger, T., & Veblen, T. T. (1997). Influences of humans and ENSO on fire history of Austrocedrus chilensis woodlands in northern Patagonia, Argentina. *Écoscience*, *4*(4), 508–520. https://doi.org/10.1080/11956860.1997.11682430

Kitzberger, T., Veblen, T. T., & Villalba, R. (1997). Climatic influences on fire regimes along a rain forest-to-xeric woodland gradient in northern Patagonia, Argentina. *Journal of Biogeography*, *24*(1), 35–47. https://doi.org/10.1111/j.1365-2699.1997.tb00048.x

Lara, A., Villalba, R., Wolodarsky-Franke, A., Aravena, J. C., Luckman, B. H., & Cuq, E. (2005). Spatial and temporal variation in Nothofagus pumilio growth at tree line along its latitudinal range (35°40′-55° S) in the Chilean Andes: Nothofagus pumilio growth at tree line. *Journal of Biogeography*, *32*(5), 879–893. https://doi.org/10.1111/j.1365-2699.2005.01191.x

Morales, M. S., Christie, D. A., Villalba, R., Argollo, J., Pacajes, J., Silva, J. S., Alvarez, C. A., Llancabure, J. C., & Soliz Gamboa, C. C. (2012). Precipitation changes in the South American Altiplano since 1300 AD reconstructed by tree-rings. *Climate of the Past*, *8*(2), 653–666. https://doi.org/10.5194/cp-8-653-2012

Moret, P., Muriel, P., Jaramillo, R., & Dangles, O. (2019). Humboldt’s Tableau Physique revisited. *Proceedings of the National Academy of Sciences*, *116*(26), 12889–12894. https://doi.org/10.1073/pnas.1904585116

Mundo, I. A., El Mujtar, V. A., Perdomo, M. H., Gallo, L. A., Villalba, R., & Barrera, M. D. (2010). Austrocedrus chilensis growth decline in relation to drought events in northern Patagonia, Argentina. *Trees*, *24*(3), 561–570. https://doi.org/10.1007/s00468-010-0427-8

Mundo, I. A., Masiokas, M. H., Villalba, R., Morales, M. S., Neukom, R., Le Quesne, C., Urrutia, R. B., & Lara, A. (2012). Multi-century tree-ring based reconstruction of the Neuquén River streamflow, northern Patagonia, Argentina. *Climate of the Past*, *8*(2), 815–829. https://doi.org/10.5194/cp-8-815-2012

Mundo, I. A., Villalba, R., Veblen, T. T., Kitzberger, T., Holz, A., Paritsis, J., & Ripalta, A. (2017). Fire history in southern Patagonia: Human and climate influences on fire activity in Nothofagus pumilio forests. *Ecosphere*, *8*(9). https://doi.org/10.1002/ecs2.1932

Muñoz, A. A., Barichivich, J., Christie, D. A., Dorigo, W., Sauchyn, D., González-Reyes, Á., Villalba, R., Lara, A., Riquelme, N., & González, M. E. (2014). Patterns and drivers of Araucaria araucana forest growth along a biophysical gradient in the northern Patagonian Andes: Linking tree rings with satellite observations of soil moisture: Patterns and drivers of Araucaria growth. *Austral Ecology*, *39*(2), 158–169. https://doi.org/10.1111/aec.12054

Rodríguez-Catón, M., Villalba, R., Srur, A., & Williams, A. P. (2019). Radial growth patterns associated with tree mortality in Nothofagus pumilio forest. *Forests*, *10*(6), 489. https://doi.org/10.3390/f10060489

Sangüesa-Barreda, G., Villalba, R., Rozas, V., Christie, D. A., & Olano, J. M. (2019). Detecting Nothofagus pumilio growth reductions induced by past spring frosts at the northern Patagonian Andes. *Frontiers in Plant Science*, *10*, 1413. https://doi.org/10.3389/fpls.2019.01413

Speziale, K. L., & Ezcurra, C. (2015). Rock outcrops as potential biodiversity refugia under climate change in North Patagonia. *Plant Ecology & Diversity*, *8*(3), 353–361. https://doi.org/10.1080/17550874.2014.983200

Suarez, M. L., Ghermandi, L., & Kitzberger, T. (2004). Factors predisposing episodic drought-induced tree mortality in Nothofagus- site, climatic sensitivity and growth trends. *Journal of Ecology*, *92*(6), 954–966. https://doi.org/10.1111/j.1365-2745.2004.00941.x

Suarez, M. L., Villalba, R., Mundo, I. A., & Schroeder, N. (2015). Sensitivity of Nothofagus dombeyi tree growth to climate changes along a precipitation gradient in northern Patagonia, Argentina. *Trees*, *29*(4), 1053–1067. https://doi.org/10.1007/s00468-015-1184-5

Urrutia‐Jalabert, R., Malhi, Y., Barichivich, J., Lara, A., Delgado‐Huertas, A., Rodríguez, C. G., & Cuq, E. (2015). Increased water use efficiency but contrasting tree growth patterns in Fitzroya cupressoides forests of southern Chile during recent decades. *Journal of Geophysical Research: Biogeosciences*, *120*(12), 2505–2524. https://doi.org/10.1002/2015JG003098

Veblen, T. T. (2010). Wildfire risk and hazard in northern Patagonia, Argentina. In M. Stoffel, M. Bollschweiler, D. R. Butler, & B. H. Luckman (Eds.), *Tree Rings and Natural Hazards* (Vol. 41, pp. 383–387). Springer Netherlands. https://doi.org/10.1007/978-90-481-8736-2_35

Veblen, T. T., Kitzberger, T., Villalba, R., & Donnegan, J. (1999). Fire history in northern Patagonia: The roles of humans and climatic variation. *Ecological Monographs*, *69*(1), 47–67. https://doi.org/10.1890/0012-9615(1999)069[0047:FHINPT]2.0.CO;2

Venegas-González, A., Juñent, F. R., Gutiérrez, A. G., Peña-Rojas, K., & Filho, M. T. (2018). Efecto de la variabilidad climática sobre los patrones de crecimiento y establecimiento de Nothofagus macrocarpa en Chile central. *Bosque (Valdivia)*, *39*(1), 81–93. https://doi.org/10.4067/S0717-92002018000100081

Venegas-González, A., Roig, F. A., Peña-Rojas, K., Hadad, M. A., Aguilera-Betti, I., & Muñoz, A. A. (2019). Recent consequences of climate change have affected tree growth in distinct Nothofagus Macrocarpa (DC.) FM Vaz & Rodr age classes in central Chile. *Forests*, *10*(8), 653. https://doi.org/10.3390/f10080653

Villalba, R. (1994). Tree-ring and glacial evidence for the medieval warm epoch and the little ice age in southern South America. *Climatic Change*, *26*(2–3), 183–197. https://doi.org/10.1007/BF01092413

Villalba, R., Boninsegna, J. A., Veblen, T. T., Schmelter, A., & Rubulis, S. (1997). Recent trends in tree-ring records from high elevation sites in the Andes of northern Patagonia. *Climatic Change*, *36*(3/4), 425–454. https://doi.org/10.1023/A:1005366317996

Villalba, R., Cook, E. R., Jacoby, G. C., D’Arrigo, R. D., Veblen, T. T., & Jones, P. D. (1998). Tree-ring based reconstructions of northern Patagonia precipitation since AD 1600. *The Holocene*, *8*(6), 659–674. https://doi.org/10.1191/095968398669095576

Villalba, R., Grau, H. R., Boninsegna, J. A., Jacoby, G. C., & Ripalta, A. (1998). Tree‐ring evidence for long‐term precipitation changes in subtropical South America. *International Journal of Climatology*, *18*(13), 1463–1478.

Villalba, R., Lara, A., Boninsegna, J. A., Masiokas, M., Delgado, S., Aravena, J. C., Roig, F. A., Schmelter, A., Wolodarsky, A., & Ripalta, A. (2003). Large-scale temperature changes across the southern Andes: 20th-century variations in the context of the past 400 Years. *Climatic Change*, *59*(1/2), 177–232. https://doi.org/10.1023/A:1024452701153

Villalba, R., Lara, A., Masiokas, M. H., Urrutia, R., Luckman, B. H., Marshall, G. J., Mundo, I. A., Christie, D. A., Cook, E. R., Neukom, R., Allen, K., Fenwick, P., Boninsegna, J. A., Srur, A. M., Morales, M. S., Araneo, D., Palmer, J. G., Cuq, E., Aravena, J. C., … LeQuesne, C. (2012). Unusual southern hemisphere tree growth patterns induced by changes in the Southern Annular Mode. *Nature Geoscience*, *5*(11), 793–798. https://doi.org/10.1038/ngeo1613

Villalba, R., & Veblen, T. T. (1997a). Spatial and temporal variation in Austrocedrus growth along the forest–steppe ecotone in northern Patagonia. *Canadian Journal of Forest Research*, *27*(4), 580–597.

Villalba, R., & Veblen, T. T. (1997b). Regional patterns of tree population age structures in northern Patagonia: Climatic and disturbance influences. *The Journal of Ecology*, *85*(2), 113. https://doi.org/10.2307/2960643

Villalba, R., & Veblen, T. T. (1998). Influences of large-scale climatic variability on episodic tree mortality in northern Patagonia. *Ecology*, *79*(8), 2624–2640. https://doi.org/10.1890/0012-9658(1998)079[2624:IOLSCV]2.0.CO;2

von Gunten, L., Grosjean, M., Rein, B., Urrutia, R., & Appleby, P. (2009). A quantitative high-resolution summer temperature reconstruction based on sedimentary pigments from Laguna Aculeo, central Chile, back to AD 850. *The Holocene*, *19*(6), 873–881. https://doi.org/10.1177/0959683609336573

Zimmer, A., Meneses, R. I., Rabatel, A., Soruco, A., Dangles, O., & Anthelme, F. (2018). Time lag between glacial retreat and upward migration alters tropical alpine communities. *Perspectives in Plant Ecology, Evolution and Systematics*, *30*, 89–102. https://doi.org/10.1016/j.ppees.2017.05.003

Table S 2 Main examples of studies that have used species distribution models (SDMs) in the Andes between 2010-2019. We conducted a search in SCOPUS using the keywords “climate change” or “warming” and “species distribution in separate queries for each Andean country (adding the country name) and one additional query with “Andes” or “Andean” and “plant”. The search was conducted to retrieve studies published from 2010, which resulted in 145 studies. We kept those records that examined plant distribution and that used SDMs, retaining 32 studies that are summarised in this table.

| **Authors** | **Title** | **Period** | **Biome/Region** | **Object of study** | **Climate data** | **Algorithm** | **Ensembling** | **Processes** | **Warming** | **Expected change under warming** |
| --- | --- | --- | --- | --- | --- | --- | --- | --- | --- | --- |
| Feeley & Silman 2010 | Modelling the responses of Andean and Amazonian plant species to climate change: The effects of georeferencing errors and the importance of data filtering | Future | High-elevation grasslands and evergreen montane forest | 1882 plant species | Elevation (upward displacement of 800 m ~ 4.5°C) | Envelope models | No | Dispersal | Yes | Mid-elevation Andean species are projected to keep their range sizes or increase it because of upward displacement. However, species with mid-elevation > 3500 m will suffer area loss. |
| Pacheco et al. 2010 | Effects of climate change on subtropical forests of South America | Future | Semideciduous montane forest areas of northern Argentina | 6 plant species | Worldclim | Maxent | No | No | Yes | Semideciduous montane forest is projected to lose about 40% of its current range |
| Soria-Auza et al. 2010 | Impact of the quality of climate models for modelling species occurrences in countries with poor climatic documentation: a case study from Bolivia | Present | Bolivian evergreen montane forest and seasonal dry forests | 22 plant species | Worldclim vs Saga | Maxent | No | No | No | - |
| Feeley et al. 2011 | Upslope migration of Andean trees | Future | Evergreen montane forest (Manu National Park, Peru) | 1000 plant species | Elevation was used for the models, but rate of future temperature change was estimated from weather stations | Envelope models | No | Dispersal | Yes | Upward migrations of 2.5-3.5 m are observed for most tropical Andean tree genera and increasing abundance of genera previously distributed at lower elevations |
| Särkinen et al. 2011 | Forgotten forests - issues and prospects in biome mapping using Seasonally Dry Tropical Forests as a case study | Present | Dry forests of South America | 1 biome | Worldclim and elevation | Maxent | No | No | No | - |
| Quiroga et al. 2012 | Shrinking forests under warming: Evidence of *Podocarpus parlatorei* (pino del cerro) from the subtropical Andes | Paleo (LGM) | Montane semi-deciduous forest | 1 plant species | Worldclim | Maxent | No | No | Climate change | No warming only LGM |
| Sede et al. 2012 | Phylogeography and palaeodistribution modelling in the Patagonian steppe: The case of *Mulinum spinosum* (Apiaceae) | Paleo (LGM and present) | Chilean and Argentinian Andean region and Patagonian steppe | 1 plant species | Worldclim | Maxent | No | No | Climate change | No warming only LGM |
| Swenson et a. 2012 | Plant and animal endemism in the eastern Andean slope: Challenges to conservation | Present | Evergreen montane forest and lowland rainforest of Peru and Bolivia. | 435 plant species | Worldclim, topographic variables and satellite derived indexes. | Maxent | No | No | No | - |
| Zutta et al. 2012 | Predicting *Polylepis* distribution: Vulnerable and increasingly important Andean woodlands | Present | Tropical high-elevation grasslands | 2 plant species | Worldclim, satellite derived products and topographic variables. | Maxent | No | No | No | - |
| Heibl & Renner 2012 | Distribution models and a dated phylogeny for Chilean Oxalis species reveal occupation  of new habitats by different lineages, not rapid adaptive radiation | Present | Atacama desert | 42 plant species | Worldclim, digital elevation model and occurrence of "fog oases" as a categorical variable. | Maxent | No | No | No | - |
| Bambach et al. 2013 | Impacts of climate change on the distribution of species and communities in the Chilean Mediterranean ecosystem | Future | Mediterranean region of Chile (sclerophyllous, woodlands and shrublands) | 14 plant species | Regional climate model for Chile (PRECIS) | Maxent | No | Dispersal | Yes | Reduction of suitable environment for all studied species (14). Highest reduction occurs along coastline, but the Andean range region has more stable habitat due to topo-climatic buffers |
| Tovar et al. 2013 | Diverging Responses of Tropical Andean Biomes under Future Climate Conditions | Future | Tropical Andean biomes | 8 biomes | Worldclim and topographic variables | Logistic regressions | No | Landuse | Yes | Important area losses being projected for high-elevation humid grasslands and evergreen montane forest, but between 74.8% and 83.1% of the Tropical Andes will remain stable. |
| Vedel-Sorensen et al. 2013 | Spatial distribution and environmental preferences of 10 economically important forest palms in western South America | Present | Tropical forests of the tropical Andes (9 palms) | 10 plant species | Worldclim, satellite derived products and soil data | Maxent | No | No | No | - |
| Nicola et al. 2014 | Geographic variation among closely related, highly variable species with a wide distribution range: The South Andean-Patagonian *Nassauvia* subgenus *Strongyloma* (Asteraceae, Nassauvieae) | Present | South Andean-Patagonia | 5 plant species | Worldclim | Maxent | No | No | No |  |
| Pliscoff et al. 2014 | Effects of alternative sets of climatic predictors on species distribution models and associated estimates of extinction risk: A test with plants in an arid environment | Future | Peruvian and Atacama deserts, and Chilean Mediterranean woodlands | 13 plant species | Faoclim dataset and 930 weather stations. | ANN, CTA, GAM, GBM, GLM, MARS, RF and SRE. | Yes | No | Yes | No significant change in distribution when averaged the 13 species located in deserts and Mediterranean woodlands. But 4 species are projected to lose area. |
| Ramirez-Villegas et al 2014 | Using species distributions models for designing conservation strategies of Tropical Andean biodiversity under climate change | Future | Tropical Andes | 9457 plant species | Worldclim | MAXENT | No | Dispersal | Yes | High elevation humid grasslands and evergreen montane forest are projected to reduce their species richness and high rates of species turnover. |
| Tejedor Garavito et al. 2015 | The relative impact of climate change on the extinction risk of tree species in the montane tropical Andes | Future | Evergreen montane forest of the Tropical Andes | 129 plant species | Worldclim | GAM, recursive partitioning (rpart) and support vector machines (KSVM). | No | No | Yes | Climate change is projected to account for a 15% increase in the overall risk of extinction of evergreen montane forest tree species. |
| Souto et al. 2015 | How do cold-sensitive species endure ice ages? Phylogeographic and paleodistribution models of postglacial range expansion of the mesothermic drought-tolerant conifer *Austrocedrus chilensis* | Paleo (LGM to present) | Mediterranean and temperate deciduous forest of the southern Andes | 1 plant species | Worldclim | Maxent | No | No | Yes | - |
| Aguirre et al. 2017 | Potential impacts to dry forest species distribution under two climate change scenarios in southern Ecuador | Future | Southern dry forest of Ecuador | 5 plant species | Worldclim | Maxent | No | No | Yes | Reduction of up to 26% in area for future distribution in dry forest (4 out of 5 species) |
| Clement et al. 2017 | Origin and dispersal of domesticated peach palm | Paleo (LGM and present) | Tropical central and south American forests | 3 varieties of one species | Worldclim (present and LGM) | Maxent | No | No | No |  |
| Cuesta et al. 2017 | Priority areas for biodiversity conservation in mainland Ecuador | Future | Mainland Ecuador | 667 plant species | Worldclim and topographic variables | Maxent | No | Landuse | Yes | 9.3% of the Ecuadorian Andes are at risk of high species turn over in scenarios of climate change. |
| Ledo & Colli 2017 | The historical connections between the Amazon and the Atlantic Forest revisited | Paleo (LIG, LGM, Mid-Holocene and present) | Tropical rainforests | 1 biome | Worldclim | Eleven algorithms of BIOMOD (best model was selected: Random Forest). | No | No | climate change | Cold adapted forest species of the Peruvian and Bolivian eastern Andes were restricted to the Andes but connected to the Atlantic forest during last interglacial (LIG). |
| Manchego et al. 2017 | Climate change versus deforestation: Implications for tree species distribution in the dry forests of southern Ecuador | Future | Dry forest of Ecuador | 17 plant species | Worldclim and topographical variables | Maxent | No | No | Yes | Climate change will result in large distributional shifts for dry forest rather than actual reduction in surface area. Land-use change is projected to have a larger effect than climate change. |
| Salariato & Zuloaga 2017 | Climatic niche evolution in the Andean genus *Menonvillea* (Cremolobeae: Brassicaceae) | Present | Argentinian and Chilean Andes | 24 plant species | Worldclim, annual aridity index and potential evapotranspiration | Maxent | No | No | No | - |
| Sosa-Pivatto et al. 2017 | Do 120,000 years of plant–pollinator interactions predict floral phenotype divergence in *Calceolaria polyrhiza*? A reconstruction using species distribution models | Paleo (LIG, LGM and present) | Patagonian steppe and Patagonian forest | 1 plant species | Worldclim | Maxent | No | Interaction | Yes | Interaction area between a plant and one bee of the Patagonian region was stable over time, including warming periods (LIG), but not for another bee (highly fragmented). |
| Wicakzono et al 2017 | Contracting montane cloud forests: a case study of the Andean alder (*Alnus acuminata*) and associated fungi in the Yungas | Future | Semi-deciduous montane forest | 1 plant species | Worldclim and soil layers | GLM, GAM and GBM | Yes | Interaction | Yes | A 20-50% or decrease in suitable area is projected for Alnus acuminata, typical of semi-deciduous montane forest, under climate change scenarios. |
| Gorostiague et al. 2018 | Will climate change cause spatial mismatch between plants and their pollinators? A test using Andean cactus species | Future | North-western Argentina | 11 plant species | Worldclim | Maxent | No | Dispersal | Yes | Projections of up to 80% range contraction for two cacti species but no spatial mismatch is expected between cacti and their pollinators under climate change scenarios. |
| Quipildor et al. 2018 | Regional climate oscillations and local topography shape genetic polymorphisms and distribution of the giant columnar cactus *Echinopsis terscheckii* in drylands of the tropical Andes | Paleo (LGM and Mid Holocene) | Tropical and subtropical dry ecotonal areas of northern and central Andes of Argentina | 1 plant species | Worldclim | Maxent | No | No | Yes | A cactus from the drylands of northwestern Argentina suffered a considerable reduction of its southern population under more humid-warm conditions (mid-Holocene). |
| Salariato et al. 2018 | Reinstatement of the Southern Andean Genus *Stenodraba* (Brassicaceae) Based on Molecular Data and Insights from its Environmental and Geographic Distribution | Present | Andes of south-central Argentina and Chile | 8 plant species | Worldclim, annual aridity index and evapotranspiration database. | Maxent | No | No | No | - |
| Fuentes-Castillo et al. 2019 | Modelling the current and future biodiversity distribution in the Chilean Mediterranean hotspot. The role of protected areas network in a warmer future | Future | Chilean Mediterranean region | 1727 plant species | Bioclimatic surfaces sourced from Pliscoff et a. 2014 | Maxent | No | No | Yes | Projected decrease of overall species richness for the Chilean Mediterranean region. |
| Mavarez et al. 2018 | Current and future distributions of Espeletiinae (Asteraceae) in the Venezuelan Andes based on statistical downscaling of climatic variables and niche modelling | Future | Paramos of Venezuela | 28 plant species | Worldclim and topographical variables | GLM, GBM, MARS, and SRE | Yes | No | Yes | Espeletiinae species, endemic to the Paramo are projected to lose between 51.3-78.1% of current distribution area under climate change scenarios. |
| Zizka et al. 2019 | Biogeography and conservation status of the pineapple family (Bromeliaceae) | Present | The Americas | 3272 plant species | CHELSA | GAM, random forest and BIOCLIM models | Yes | No | No | - |

**References**

Aguirre, N., Eguiguren, P., Maita, J., Ojeda, T., Sanamiego, N., Furniss, M. & Aguirre, Z. (2017) Potential impacts to dry forest species distribution under two climate change scenarios in southern Ecuador. *Neotropical Biodiversity*, **3**, 18–29.

Bambach, N., Meza, F.J., Gilabert, H. & Miranda, M. (2013) Impacts of climate change on the distribution of species and communities in the Chilean Mediterranean ecosystem. *Regional Environmental Change*, **13**, 1245–1257.

Clement, C.R., Cristo-Araújo, M. de, Coppens d’Eeckenbrugge, G., Reis, V.M. dos, Lehnebach, R. & Picanço-Rodrigues, D. (2017) Origin and dispersal of domesticated peach palm. *Frontiers in Ecology and Evolution*, **5**, 148.

Cuesta, F., Peralvo, M., Merino-Viteri, A., Bustamante, M., Baquero, F., Freile, J.F., Muriel, P. & Torres-Carvajal, O. (2017) Priority areas for biodiversity conservation in mainland Ecuador. *Neotropical Biodiversity*, **3**, 93–106.

Feeley, K.J. & Silman, M.R. (2011) Keep collecting: accurate species distribution modelling requires more collections than previously thought: Temporal autocorrelated biases necessitate more collections. *Diversity and Distributions*, **17**, 1132–1140.

Feeley, K.J. & Silman, M.R. (2010) Land-use and climate change effects on population size and extinction risk of Andean plants. *Global Change Biology*, **16**, 3215–3222.

Fuentes‐Castillo, T., Scherson, R.A., Marquet, P.A., Fajardo, J., Corcoran, D., Román, M.J. & Pliscoff, P. (2019) Modelling the current and future biodiversity distribution in the Chilean Mediterranean hotspot. The role of protected areas network in a warmer future. *Diversity and Distributions*, **25**, 1897–1909.

Gorostiague, P., Sajama, J. & Ortega-Baes, P. (2018) Will climate change cause spatial mismatch between plants and their pollinators? A test using Andean cactus species. *Biological Conservation*, **226**, 247–255.

Heibl, C. & Renner, S.S. (2012) Distribution models and a dated phylogeny for Chilean Oxalis species reveal occupation of new habitats by different lineages, not papid adaptive radiation. *Systematic Biology*, **61**, 823–834.

Ledo, R.M.D. & Colli, G.R. (2017) The historical connections between the Amazon and the Atlantic Forest revisited. *Journal of Biogeography*, **44**, 2551–2563.

Manchego, C.E., Hildebrandt, P., Cueva, J., Espinosa, C.I., Stimm, B. & Günter, S. (2017) Climate change versus deforestation: Implications for tree species distribution in the dry forests of southern Ecuador. *PLOS ONE*, **12**, e0190092.

Mavárez, J., Bézy, S., Goeury, T., Fernández, A. & Aubert, S. (2018) Current and future distributions of Espeletiinae (Asteraceae) in the Venezuelan Andes based on statistical downscaling of climatic variables and niche modelling. *Plant Ecology & Diversity*, 1–15.

Nicola, M.V., Johnson, L.A. & Pozner, R. (2014) Geographic variation among closely related, highly variable species with a wide distribution range: the South Andean-Patagonian *Nassauvia* subgenus *Strongyloma* (Asteraceae, Nassauvieae). *Systematic Botany*, **39**, 331–348.

Pacheco, S., Malizia, L.R. & Cayuela, L. (2010) Effects of climate change on subtropical forests of South America. *Tropical Conservation Science*, **3**, 423–437.

Pliscoff, P., Luebert, F., Hilger, H.H. & Guisan, A. (2014) Effects of alternative sets of climatic predictors on species distribution models and associated estimates of extinction risk: A test with plants in an arid environment. *Ecological Modelling*, **288**, 166–177.

Quipildor, V.B., Kitzberger, T., Ortega-Baes, P., Quiroga, M.P. & Premoli, A.C. (2018) Regional climate oscillations and local topography shape genetic polymorphisms and distribution of the giant columnar cactus *Echinopsis terscheckii* in drylands of the tropical Andes. *Journal of Biogeography*, **45**, 116–126.

Quiroga, M.P., Pacheco, S., Malizia, L.R. & Premoli, A.C. (2012) Shrinking Forests under Warming: Evidence of *Podocarpus parlatorei* (pino del cerro) from the subtropical Andes. *Journal of Heredity*, **103**, 682–691.

Ramirez-Villegas, J., Cuesta, F., Devenish, C., Peralvo, M., Jarvis, A. & Arnillas, C.A. (2014) Using species distributions models for designing conservation strategies of Tropical Andean biodiversity under climate change. *Journal for Nature Conservation*, **22**, 391–404.

Salariato, D.L., Al-Shehbaz, I.A. & Zuloaga, F.O. (2018) Reinstatement of the southern Andean genus *Stenodraba* (Brassicaceae) based on molecular data and insights from its environmental and geographic distribution. *Systematic Botany*, **43**, 35–52.

Salariato, D.L. & Zuloaga, F.O. (2017) Climatic niche evolution in the Andean genus *Menonvillea* (Cremolobeae: Brassicaceae). *Organisms Diversity & Evolution*, **17**, 11–28.

Särkinen, T., Iganci, J.R., Linares-Palomino, R., Simon, M.F. & Prado, D.E. (2011) Forgotten forests - issues and prospects in biome mapping using Seasonally Dry Tropical Forests as a case study. *BMC Ecology*, **11**, 27.

Sede, S.M., Nicola, M.V., Pozner, R. & Johnson, L.A. (2012) Phylogeography and palaeodistribution modelling in the Patagonian steppe: the case of *Mulinum spinosum* (Apiaceae): Phylogeography and palaeodistribution modelling of *Mulinum spinosum*. *Journal of Biogeography*, **39**, 1041–1057.

Soria-Auza, R.W., Kessler, M., Bach, K., Barajas-Barbosa, P.M., Lehnert, M., Herzog, S.K. & Böhner, J. (2010) Impact of the quality of climate models for modelling species occurrences in countries with poor climatic documentation: a case study from Bolivia. *Ecological Modelling*, **221**, 1221–1229.

Sosa-Pivatto, M., Cosacov, A., Baranzelli, M.C., Iglesias, M.R., Espíndola, A. & Sérsic, A.N. (2017) Do 120,000 years of plant–pollinator interactions predict floral phenotype divergence in *Calceolaria polyrhiza*? A reconstruction using species distribution models. *Arthropod-Plant Interactions*, **11**, 351–361.

Souto, C.P., Kitzberger, T., Arbetman, M.P. & Premoli, A.C. (2015) How do cold-sensitive species endure ice ages? Phylogeographic and paleodistribution models of postglacial range expansion of the mesothermic drought-tolerant conifer *Austrocedrus chilensis*. *New Phytologist*, **208**, 960–972.

Swenson, J.J., Young, B.E., Beck, S., Comer, P., Córdova, J.H., Dyson, J., Embert, D., Encarnación, F., Ferreira, W., Franke, I., Grossman, D., Hernandez, P., Herzog, S.K., Josse, C., Navarro, G., Pacheco, V., Stein, B.A., Timaná, M., Tovar, A., Tovar, C., Vargas, J. & Zambrana-Torrelio, C.M. (2012) Plant and animal endemism in the eastern Andean slope: challenges to conservation. *BMC Ecology*, **12**, 1.

Tejedor Garavito, N., Newton, A.C., Golicher, D. & Oldfield, S. (2015) The relative impact of climate change on the extinction risk of tree species in the montane tropical Andes. *PLOS ONE*, **10**, e0131388.

Tovar, C., Arnillas, C.A., Cuesta, F. & Buytaert, W. (2013) Diverging responses of tropical Andean biomes under future climate conditions. *PLoS ONE*, **8**, e63634.

Vedel-Sørensen, M., Tovaranonte, J., Bøcher, P.K., Balslev, H. & Barfod, A.S. (2013) Spatial distribution and environmental preferences of 10 economically important forest palms in western South America. *Forest Ecology and Management*, **307**, 284–292.

Wicaksono, C.Y., Aguirre-Guiterrez, J., Nouhra, E., Pastor, N., Raes, N., Pacheco, S. & Geml, J. (2017) Contracting montane cloud forests: a case study of the Andean alder (*Alnus acuminata*) and associated fungi in the Yungas. *Biotropica*, **49**, 141–152.

Zizka, A., Azevedo, J., Leme, E., Neves, B., Costa, A.F., Caceres, D. & Zizka, G. (2020) Biogeography and conservation status of the pineapple family (Bromeliaceae). *Diversity and Distributions*, **26**, 183–195.

Zutta, B., R., Rundell, P.W., Saatchi, S., Casana, J.D., Gauthier, P., Soto, A., Velazco, Y. & Buermann, W. (2012) Prediciendo la distribución de *Polylepis*: bosques Andinos vulnerables y cada vez más importantes. *Revista Peruana de Biología*, **19**, 205–21

Table S 3 CMIP5 models and variables used in this study. The horizontal resolution of each model is indicated for both latitudinal and longitudinal directions (in degrees). Pr is precipitation while Tn and Tx are the minimum and maximum near-surface temperature, respectively.

| **Models** | **Lon res (°)** | **Lat res. (°)** | **Variables** | **Projected changes in climate** | **Projected changes in biomes** |
| --- | --- | --- | --- | --- | --- |
| ACCESS1-0 | 1.875 | 1.25 | Pr, Tn, Tx | X | x |
| ACCESS1-3 | 1.875 | 1.25 | Pr, Tn, Tx | X | x |
| Bcc-csm1-1 | 2.8125 | 2.78938 | Pr, Tn, Tx | X | x |
| BNU-ESM | 2.8125 | 2.78938 | Pr, Tn, Tx | X | x |
| CanESM2 | 2.8125 | 2.78938 | Pr, Tn, Tx | X | x |
| CNRM-CM5-2 | 1.40625 | 1.40013 | Pr, Tn, Tx | X |  |
| CNRM-CM5 | 1.40625 | 1.40013 | Pr, Tn, Tx | X | x |
| CSIRO-Mk3-6-0 | 1.875 | 1.86396 | Pr, Tn, Tx | X | x |
| GFDL-CM2p1 | 2.5 | 2.02247 | Pr | X |  |
| GFDL-CM3 | 2.5 | 2 | Pr, Tn, Tx | X | x |
| GFDL-ESM2G | 2.5 | 2.02247 | Pr, Tn, Tx | X | x |
| GFDL-ESM2M | 2.5 | 2.02247 | Pr, Tn, Tx | X | x |
| GISS-E2-H-CC | 2.5 | 2 | Pr, Tn, Tx | X | x |
| GISS-E2-H | 2.5 | 2 | Pr, Tn, Tx | X | x |
| GISS-E2-R | 2.5 | 2 | Pr, Tn, Tx | X | x |
| HadGEM2-AO | 1.875 | 1.25 | Pr, Tn, Tx | X |  |
| HadGEM2-CC | 1.875 | 1.25 | Pr, Tn, Tx | X | x |
| HadGEM2-ES | 1.875 | 1.25 | Pr, Tn, Tx | X | x |
| Inmcm4 | 2 | 1.5 | Pr, Tn, Tx | X | x |
| IPSL-CM5A-LR | 3.75 | 1.89474 | Pr, Tn, Tx | X | x |
| IPSL-CM5A-MR | 2.5 | 1.26761 | Pr, Tn, Tx | X | x |
| IPSL-CM5B-LR | 3.75 | 1.89474 | Pr, Tn, Tx | X | x |
| MIROC5 | 2.8125 | 2.7893 | Pr, Tn, Tx | X | x |
| MIROC-ESM | 2.8125 | 2.7893 | Pr, Tn, Tx | X |  |
| MPI-ESM-LR | 1.875 | 1.86396 | Pr, Tn, Tx | X | x |
| MPI-ESM-MR | 1.875 | 1.865185 | Pr, Tn, Tx | X | X |
| MRI-CGCM3 | 1.125 | 1.12133 | Pr, Tn, Tx | X | x |
| MRI-ESM1 | 1.125 | 1.12133 | Pr, Tn, Tx | X | x |
| NorESM1-ME | 2.5 | 1.89474 | Pr | X |  |
| NorESM1-M | 2.5 | 1.89474 | Pr, Tn, Tx | X | x |

Table S 4 Locations (bounding boxes of 2°x2° lat/long size) for which projected climate change was analysed across the Andes. The X and Y in the table represent the coordinates in decimal degrees of the top left corner of the squares in Fig. 1a.

| **ID** | **Country** | **Full name** | **Short name** | **Longitude** | **Latitude** | **Main Andean vegetation types (local names)** |
| --- | --- | --- | --- | --- | --- | --- |
| 1 | Venezuela | Mérida | Mérida | -70.97 | 10.54 | Bosque montano, Páramo andino and Bosque seco |
| 2 | Colombia | Bucaramanga-Cocuy | Bucaramanga-Cocuy | -74 | 7.5 | Bosque montano, páramo andino |
| 3 | Colombia | Cali-Nevado del Huila | Cali-Huila | -77.1 | 4.35 | Bosque montano, páramo andino |
| 4 | Ecuador | Quito | Quito | -79.29 | 0.64 | Bosque montano, páramo andino |
| 5 | Ecuador-Peru | Loja | Loja | -80.24 | -3.13 | Bosque montano, Bosque seco |
| 6 | Peru | Parques Nacionales Abiseo-Huascarán | Abiseo-Huascarán | -78.5 | -7.5 | Puna húmeda, Prepuna, Yungas Peruanas |
| 7 | Peru | Lima-Junín | Lima-Junín | -76.75 | -11 | Puna húmeda, Prepuna, Yungas Peruanas |
| 8 | Peru | Cotahuasi | Cotahuasi | -73.4 | -14 | Puna húmeda, Puna seca |
| 9 | Peru-Bolivia | Titicaca-Madidi | Titicaca-Madidi | -70 | -14 | Puna húmeda, Vegetación altoandina, Yungas Bolivianas |
| 10 | Bolivia | Salar de Uyuni | Uyuni | -69 | -18.25 | Puna seca |
| 11 | Bolivia | Sucre-Potosí | Sucre-Potosí | -65.75 | -18.25 | Chaco serrano, Bosque Boliviano-Tucumano |
| 12 | Argentina | Salta-Jujuy | Salta-Jujuy | -66.88 | -23.77 | Altoandino, Puna, Prepuna, Yungas Argentinas |
| 13 | Argentina | Tinogasta | Tinogasta | -68.76 | -27 | Altoandino, Prepuna, Monte de sierras y bolsones, Monte |
| 14 | Chile-Argentina | Aconcagua-Reserva Chinchillas | Aconcagua | -71.5 | -30.9 | Estepa altoandina, Vegas altoandinas |
| 15 | Chile-Argentina | Reserva Nacional Lircay - Río Atuel | Lircay-Atuel | -71.5 | -34.76 | Bosque andino patagónico dominado por árboles deciduos |
| 16 | Chile-Argentina | Parques Nacionales Villarrica-Lanín | Villarica-Lanín | -72.5 | -38.5 | Bosque andino patagónico dominado por árboles deciduos, Estepa altoandina, Monte |
| 17 | Chile-Argentina | Parques Nacionales Corcovado-Alerces | Corcovado-Alerces | -72.91 | -41.7 | Bosque andino patagónico dominado por coníferas |
| 18 | Chile | Parque Nacional Laguna San Rafael | San Rafael | -73.64 | -45.8639 | Bosque siempreverde montano, Turberas |
| 19 | Chile | Bernardo O'Higgins | O'Higgins | -75.5 | -49 | Bosque andino patagónico |

Table S 5 Andean biomes. Three maps (Luebert, Federico & Pliscoff, 2018; Oyarzabal et al., 2018; Tovar et al., 2013) were merged to obtain the Andean biomes map. The categories of each map were standardised and merged by considering vegetation types and climate, leaf type, and leaf phenology to characterize the predominant environmental conditions. Additionally, we added an elevation/geographical category. Local names for these regions are also shown.

| **Biome** | **Climate** | **Leaf type** | **Elevation category** | **Geography** | **Vegetation types** | **Local name of vegetation types encompassed in the biome** |
| --- | --- | --- | --- | --- | --- | --- |
| Dry shrublands and forests | dry | deciduous | middle and lower Andes | central Andes | shrubland and forest | Monte de sierras y bolsones, Prepuna |
| Temperate coniferous forests | temperate |  | middle Andes | southern Andes | forest | Bosque andino patagónico dominado por coníferas |
| Mediterranean deciduous forests | mediterranean | deciduous | middle Andes | southern Andes | forest | Bosque caducifolio |
| Temperate deciduous forests | temperate | deciduous | middle Andes | southern Andes | forest | Bosque andino patagónico dominado por árboles deciduos |
| Dry forests | dry | semi deciduous | middle Andes | northern and central Andes | forest | Chaco Serrano de Argentina, Bosque seco chiquitano, Bosque Seco de Ecuador y Perú, Valles secos de los valles del Apurímac, Marañón (Perú) |
| Semideciduous montane forests | humid | semi deciduous | middle Andes | central Andes | forest | Selvas subtropicales de montaña, Yungas de Argentina, Selva Tucumano Boliviana |
| Evergreen montane forests | humid | evergreen | middle Andes | northern and Central Andes | forest | Bosques nublados, Yungas Bolivianas y Peruanas |
| Temperate evergreen forests | temperate | evergreen | middle Andes | southern Andes | Forest | Bosque Valdiviano, Bosque patagónico dominado por árboles siempreverdes |
| Mediterranean sclerophyllous scrublands and forests | mediterranean | sclerophyllous | lower Andes | southern Andes | scrubland and forest | Bosque y matorral esclerófilo (Chile), Matorral espinoso (Chile) |
| Humid high Andean steppes | humid |  | higher Andes | southern Andes | steppe | Estepa altoandina |
| Dry high Andean steppes | dry |  | higher Andes | central and southern Andes | steppe | Puna seca (Perú y Bolivia), Puna and Altoandino (Argentina) |
| Patagonian steppes | dry |  | lower Andes | southern Andes | steppe | Estepa patagónica |
| Humid high Andean grassland and shrublands | humid |  | higher Andes | central Andes | grassland and shrubland | Puna húmeda (Perú y Bolivia) |
| Very humid high Andean grassland and shrublands | very humid |  | higher Andes | northern Andes | grassland and shrubland | Páramos |
| Peatlands | very humid |  | lower Andes | southern Andes | peatland/grassland | Mallines y Turberas al extremo sur de Patagonia |

**References**

Luebert, Federico, & Pliscoff, P. A. (2018). Sinopsis bioclimática y vegetacional de Chile (2nd ed.). Editorial Universitaria.

Oyarzabal, M., Clavijo, J., Oakley, L., Biganzoli, F., Tognetti, P., Barberis, I., Maturo, H. M., Aragón, R., Campanello, P. I., Prado, D., Oesterheld, M., & León, R. J. C. (2018). Unidades de vegetación de la Argentina. Ecología Austral, 28(1), 040–063. https://doi.org/10.25260/EA.18.28.1.0.399

Tovar, C., Arnillas, C. A., Cuesta, F., & Buytaert, W. (2013). Diverging responses of tropical Andean biomes under future climate conditions. PLoS ONE, 8(5), e63634.
